# Do Personalized Nutrition Interventions Improve Dietary Intake and Risk Factors in Adults With Elevated Cardiovascular Disease Risk Factors? A Systematic Review and Meta-analysis of Randomized Controlled Trials

**DOI:** 10.1093/nutrit/nuae149

**Published:** 2024-10-17

**Authors:** Victoria Cross, Jordan Stanford, María Gómez-Martín, Clare E Collins, Seaton Robertson, Erin D Clarke

**Affiliations:** School of Health Sciences, College of Health, Medicine and Wellbeing, The University of Newcastle, Callaghan, NSW 2308, Australia; School of Health Sciences, College of Health, Medicine and Wellbeing, The University of Newcastle, Callaghan, NSW 2308, Australia; Food and Nutrition Research Program, Hunter Medical Research Institute, New Lambton Heights, NSW 2305, Australia; School of Health Sciences, College of Health, Medicine and Wellbeing, The University of Newcastle, Callaghan, NSW 2308, Australia; Food and Nutrition Research Program, Hunter Medical Research Institute, New Lambton Heights, NSW 2305, Australia; School of Health Sciences, College of Health, Medicine and Wellbeing, The University of Newcastle, Callaghan, NSW 2308, Australia; Food and Nutrition Research Program, Hunter Medical Research Institute, New Lambton Heights, NSW 2305, Australia; School of Health Sciences, College of Health, Medicine and Wellbeing, The University of Newcastle, Callaghan, NSW 2308, Australia; School of Health Sciences, College of Health, Medicine and Wellbeing, The University of Newcastle, Callaghan, NSW 2308, Australia; Food and Nutrition Research Program, Hunter Medical Research Institute, New Lambton Heights, NSW 2305, Australia

**Keywords:** cardiovascular disease, personalized nutrition, medical nutrition therapy, risk factors, diet

## Abstract

**Context:**

Dietary modifications can improve cardiovascular disease (CVD) risk factors. Personalized nutrition (PN) refers to individualized nutrition care based on genetic, phenotypic, medical, behavioral, and/or lifestyle characteristics. PN may be beneficial in improving CVD risk factors, including diet. However, this has not been reviewed previously.

**Objective:**

The aim was to evaluate the effectiveness of PN interventions on CVD risk factors and diet in adults at elevated CVD risk.

**Data Sources:**

Six databases were searched for randomized controlled trials published between 2000 and 2023 that tested the impact of PN interventions on CVD risk factors in people at elevated risk.

**Data Extraction:**

Risk of bias was assessed using the Academy of Nutrition and Dietetics Quality Criteria checklist. Data synthesis of eligible articles included participant characteristics, intervention details, and change in primary CVD risk factor outcomes, including blood pressure (BP), plasma lipids, and CVD risk score, and secondary risk factors, including anthropometric outcomes and diet quality. Random-effects meta-analyses were conducted to explore weighted mean differences (WMDs) in change or final mean values for studies with comparable data (studies with dietary counseling interventions) for outcomes including BP, blood lipids, and anthropometric measurements.

**Data Analysis:**

Of 7676 identified articles, 16 articles representing 15 studies met the inclusion criteria. Studies included between 40 and 563 participants and reported outcomes for CVD risk factors, including hyperlipidemia (n = 5), elevated BP (n = 3), overweight/obesity (n = 1), and multiple risk factors (n = 6). Risk of bias was low. Results suggested potential benefit of PN on systolic BP (WMD: −1.91; 95% CI: −3.51, −0.31 mmHg) and diastolic BP (WMD: −1.49; 95% CI: −2.39, −0.58 mmHg) and dietary intake in individuals at high CVD risk. Results were inconsistent for plasma lipid and anthropometric outcomes.

**Conclusion:**

Results were promising for PN interventions that used dietary counseling on CVD risk factors in at-risk individuals. However, further evidence for other personalization methods is required, including improving methodological quality and longer study duration in future PN interventions.

**Systematic Review Registration:**

OpenScience Framework (https://doi.org/10.17605/OSF.IO/SHVWP).

## INTRODUCTION

Cardiovascular disease (CVD) spans a group of disorders of heart and blood vessels, including coronary heart and cerebrovascular disease.^1^ Cardiovascular disease is the leading cause of death worldwide.^1^ In 2019, CVD was the underlying cause of 32% of deaths, and responsible for 393 million lost years of healthy life globally.^2^^,^^3^ In addition, CVD contributes to rising global economic burden, estimated at $863 billion US dollars (USD) in 2010, and is expected to increase to $1044 billion USD by 2030.^4^ Approximately 70% of CVD burden is attributed to modifiable risk factors, such as high blood pressure (BP), dietary risks, overweight/obesity, and tobacco use.^5^ As poor diet quality is a major contributor to CVD burden, guidelines recommend dietary intervention as a first-line approach to the prevention and management of CVD, including reducing consumption of saturated fat and sodium, increasing consumption of fruit, vegetables, whole grains, and omega-3 fatty acid–rich foods, as well as adherence to whole dietary patterns such as the Dietary Approaches to Stop Hypertension (DASH) and Mediterranean diets.^6^^,^^7^

Evidence-based recommendations support referral to a dietitian for medical nutrition therapy (MNT) for management of various CVD risk factors, including obesity, hypertension, and dyslipidemia.^8–10^ For individuals evaluated as being at high CVD risk, frequent, sustained, and specific dietary advice is recommended.^11^ Previous systematic reviews have reported benefits of MNT provided by dietitians in individuals at elevated CVD risk for risk factors including blood lipids (total cholesterol [TC]; mean difference [MD]: 95% CI: −20.84 mg/dL; *P* = .04; low-density-lipoprotein cholesterol [LDL-C]; −11.56 mg/dL; *P* = .02; and triglycerides [TG]; −32.55 mg/dL; *P* = .01),^12^^,^^13^ BP (systolic BP [SBP]; MD: −3.04 mmHg; *P* = .079; diastolic BP [DBP]; −1.99 mmHg; *P* = .011),^14^ and overweight/obesity (body mass index [BMI]; MD: −1.5 kg/m^2^).^14^

Personalized nutrition (PN) broadly describes the tailoring of specific nutritional guidance to an individual’s characteristics, including genetic or phenotypic data, medical history, dietary intake or nutritional status, and/or behavioral characteristics.^15^^,^^16^ It is proposed that using PN to target nutrition services based on these, often complex, individual determinants of dietary behavior can be more effective in facilitating lasting dietary behavior change compared with generic, population-level nutrition advice.^17^ Personalized nutrition shares similar, often overlapping, meanings with terms such as “individualized nutrition,” “precision nutrition,” and “nutrigenomics” throughout the literature.^15^ Although these terms are not currently clearly defined, there is overall agreement that the goal of PN is to advance human health and well-being by tailoring nutrition recommendations and interventions to an individual or group of individuals with similar traits.^15^ Medical nutrition therapy delivered by dietitians can be characterized as a type of PN.^18^

A 2021 systematic review of randomized controlled trials (RCTs) of PN interventions in healthy participants with differing diet, phenotypic, and genotypic information identified PN to be effective in improving overall dietary patterns, as well as in lowering total energy, fat, saturated fat, and sodium intakes.^19^ Two included studies reported greater improvements in CVD risk factors in participants who received PN advice compared with those receiving usual care, including for LDL-C (92.3 ± 32.9 mg/dL vs 105.9 ±33.3 mg/dL; *P* = .03),^20^ BMI (mean difference: −1.1 kg/m^2^ vs −0.1 kg/m^2^), waist circumference (mean difference: −5.0 cm vs −4.2 cm), and BP (mean difference: SBP: −10.6 mmHg vs −4.5 mmHg; DBP: −2.9 mmHg vs −0.7 mmHg). However, 1 study did not find a statistically significant difference in the results.^21^

While these results are promising, no previous systematic reviews have specifically examined the effects of PN on a variety of measures of CVD risk, nor have any examined populations with different CVD risk factors. Therefore, the aim of the current systematic review was to synthesize evidence of the effectiveness of PN on CVD risk factors and dietary outcomes in people at elevated CVD risk. We hypothesized that PN interventions will result in greater improvements in CVD risk factors, such as plasma cholesterol, BP, anthropometric outcomes, and dietary intake, when compared with standard care or no intervention.

## METHODS

### Study Design

This systematic review and meta-analysis followed the Preferred Reporting Items for Systematic Reviews and Meta-Analyses (PRISMA) guidelines (Table S1).^22^ The current systematic review was registered with OpenScience Framework (https://doi.org/10.17605/OSF.IO/SHVWP).^23^

### Eligibility Criteria

To be eligible for inclusion, articles were required to meet predefined Population, Intervention, Control, Outcome, and Study design (PICOS) criteria (Table 1). Included studies were those conducted in participants at an increased risk of CVD, determined by anthropometric measures such as overweight, obesity, or BMI ≥25 kg/m^2^; clinical indicators such as dyslipidemia, high BP, calculated genetic risk, or high overall CVD risk scores including Framingham Risk Score (FRS) or Absolute CVD Risk Score or the equivalent, utilized for the estimation of an individual's likelihood of developing CVD within a specified timeframe.^11^^,^^24^ Studies were limited to those published after 2000 to ensure that only the most up-to-date studies were captured.

**Table 1.** PICOS (Population, Intervention, Comparison, Outcome. and Study design) Criteria for Inclusion and Exclusion of Studies

Parameter	Inclusion criteria	Exclusion criteria
Population	Adult participants (>18 y) at elevated risk of cardiovascular disease (CVD)	Participants who are pregnant or have a history of gestational hypertension or preeclampsia
Intervention/exposure	Studies investigating a personalized or precision nutrition intervention	Supplement-based trials, studies in acute care or inpatient settings
Comparison	Usual care or no intervention	Other intervention arms including supplement- or pharmacological-based trials outside of usual care; otherwise, personalized lifestyle interventions
Outcome	Changes in CVD risk factors (including blood lipids, blood pressure, genetic risk score, absolute risk score, or a cardiovascular-related event). Secondary outcomes included dietary intake (eg, dietary patterns, diet quality) and anthropometric outcomes (eg, weight, body mass index [BMI]).	Any outcomes not listed in the inclusion criteria
Study design	Randomized controlled trials	All other study designs

### Search Strategy

Six electronic databases were searched on April 16, 2023 (Table S2). The search strategy was designed with the assistance of an experienced health research librarian and was divided into 6 groups: (1) heart disease, cholesterol, hyperlipidaemia, hypercholesterolaemia, BP, cardiovascular disease; (2) personalised/individualised diet, nutrigenomics, genomics, MNT/diet therapy, counsel; (3) diet, nutrition; (4) effect, outcome change; (5) recommendation, advice, program, counsel, intervention; (6) RCT, randomised intervention. Articles were limited to include only RCTs, human beings, adults (>18 years), and articles published in the English language (Table S2).

### Study Selection

All studies identified were retrieved and duplicates were removed using the Systematic Review Accelerator Deduplicator tool^25^ and uploaded to Covidence version 2.0 (Veritas Health Innovation, Melbourne, Australia) where any additional duplicates were removed.^26^ Titles and abstracts were screened by 2 independent reviewers against inclusion and exclusion criteria to determine whether full texts should be retrieved. Two reviewers then undertook screening of full texts to determine if they met eligibility criteria. Conflicts between reviewers that could not be resolved via group discussion were evaluated by a third independent reviewer.

### Data Extraction

Data were extracted from eligible studies by 1 author using a standardized tool, with key data reported as follows: stated aim; setting; intervention description (method of personalization [eg, MNT from an accredited practicing dietitian (APD) or equivalent, genetics], intensity, and duration); participant characteristics including age, sex, country, health condition; outcome measures (eg, clinical indicators [BP]; serum measures including lipids; anthropometric measures [weight, BMI, waist circumference] and dietary intake); comparator description; and key findings. The data were then extracted by the first reviewer and cross-checked by a second reviewer.

### Quality Assessment and Risk of Bias

Study quality was assessed in duplicate using the Academy of Nutrition and Dietetics Quality Criteria Tool.^27^ The quality of each study was evaluated using a checklist comprising 10 criteria, evaluating aspects such as the research question, group selection process, comparability, withdrawals, interventions, outcomes, statistical analysis, conclusions, and conflicts of interest. The checklist used a binary “yes” or “no” format to categorize each article as negative, neutral, or positive. Articles receiving “no” responses to 6 or more questions were classified as negative. If most questions received “yes” responses, with specific criteria such as selection process, group comparability, interventions, and outcomes all receiving a “yes” and at least 1 other criterion classified as “yes,” then the article was designated as positive. Articles that fell between the 2 ratings were classified as neutral. Study quality was assessed by 1 reviewer (V.C.) and 33% were cross-checked by a second independent reviewer (E.D.C., J.S., C.E.C., or M.G.-M.). Any discrepancies were resolved through discussion and consensus.

### Data Synthesis

Data were synthesized narratively and organized according to primary CVD risk factor, intervention and comparator treatments, and outcome measures grouped as clinical outcomes, anthropometric outcomes, and dietary outcomes.

Random-effects meta-analysis was conducted using Review Manager software (RevMan version 5.4.1; Copenhagen: The Nordic Cochrane Centre, The Cochrane Collaboration).^28^ Due to variation in intervention characteristics and outcomes, synthesis via meta-analysis was only deemed appropriate for studies that included a dietary counseling intervention component and reported cardiometabolic and/or anthropometric outcomes. Furthermore, outcomes were only included in the meta-analysis if they were evaluated in 3 or more studies. Mean changes in relevant outcomes were extracted where possible, and study authors were contacted to confirm or retrieve data if the published article did not contain sufficient information for meta-analysis. When these data were not available, mean final values were retrieved and included as a separate subgroup.^29^ Where units were varied between studies, data were converted into common units. For example, where plasma lipid concentrations were expressed in mg/dL, this was converted to mmol/L by multiplying by 0.0113 for TG and by 0.0259 for cholesterol.^30^ Standard deviations were converted from standard errors or 95% CIs using formulas in the Cochrane Handbook.^29^ The proportion of total variation attributable to between-study heterogeneity was estimated using the *I*^2^ statistic for each analysis. An *I*^2^ value ≥75% was considered a high level of inconsistency, 50%–75% was considered substantial, 36%–60% was moderate, and 0%–35% was considered low inconsistency as recommended by Higgins and Thompson.^31^ If a study had multiple relevant intervention arms, or outcome data were otherwise divided (eg, participants separated based on sex in Lim et al^32^), results were combined using equations from the Cochrane Handbook.^29^ Subgroup analysis was performed examining dietitian-led studies alone by excluding non–dietitian-led studies from each outcome. Sensitivity analysis was performed by excluding each article sequentially to explore the impact of each individual study on the overall effect.

## RESULTS

**Figure 1.**
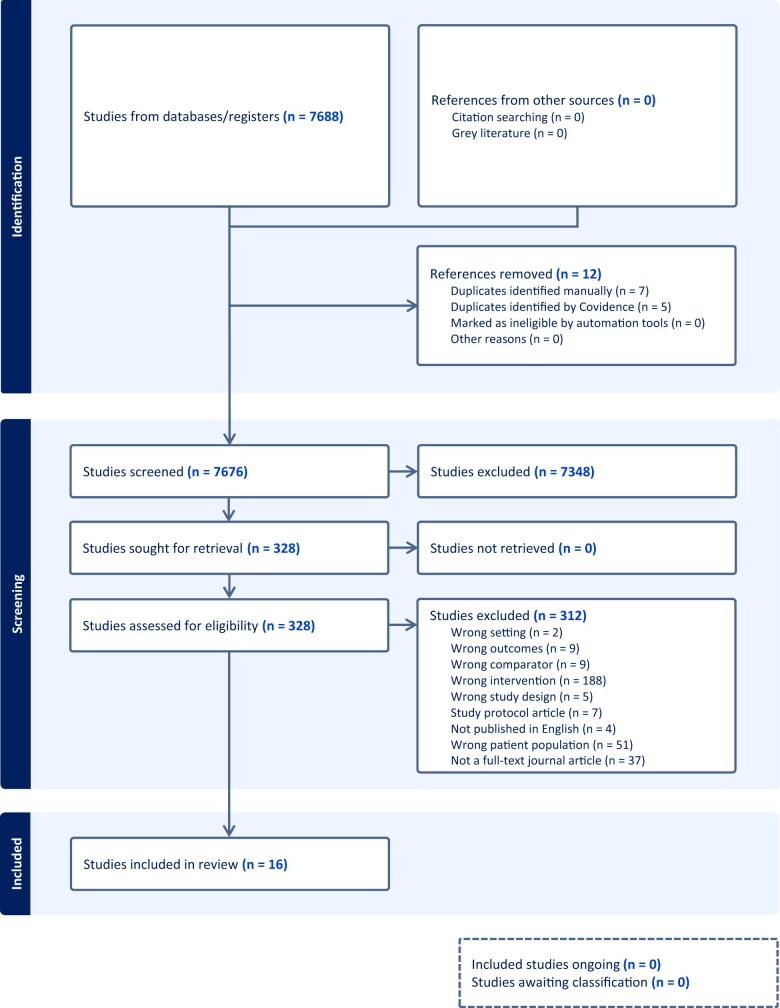
PRISMA (Preferred Reporting Items for Systematic Reviews and Meta-Analyses) Flow Chart

As detailed in Figure 1, a total of 7676 studies were identified using the search strategy. After title and abstract screening, 328 full-text articles were reviewed. A final 16 articles^32–47^ reporting data on 15 unique studies met the predetermined inclusion criteria and were included in this review. The articles by Wong et al (2015)^46^ and Wong et al (2016)^47^ were the same intervention and are summarized together with the characteristics presented as Wong et al (2015).^46^ The primary reasons for exclusion were incorrect intervention, including non-personalized dietary interventions (60%) or comprehensive lifestyle interventions, and wrong patient population (16%) including individuals not at elevated risk of CVD.

### Characteristics of Included Studies

Characteristics of included studies are detailed in Table S3. In summary, all 15 included studies were parallel RCTs. The sample size of participants ranged from 40^32^ to 563,^39^ with an age range from 18 to 89 years. One study included male participants only,^39^ while all other studies included both females and males. Five studies were based in North America,^34–37^^,^^42^ 6 were based in Asia,^32^^,^^38^^,^^41^^,^^43^^,^^46^^,^^47^ and 5 were based in Europe.^33^^,^^39^^,^^40^^,^^44^^,^^45^ Studies included participants who had different risk factors for CVD. As outlined in Table S3, these risk factors consisted of hyperlipidemia (*n* = 5),^32^^,^^34^^,^^36^^,^^38^^,^^44^ high BP (*n* = 3),^42^^,^^46^^,^^47^ and overweight/obesity (*n* = 1).^33^ A further 7 studies included participants with a combination of risk factors—for example, high SBP and elevated BMI (>26 kg/m^2^).^35^^,^^37^^,^^39–41^^,^^43^^,^^45^

### Quality Assessment of Studies

The quality of included studies is presented in Table S4. All 16 articles were allocated an overall positive rating using the quality criteria checklist. Despite overall positive ratings, some sections within the quality assessments were generally scored more negatively across studies. These included that the majority of studies did not involve blinding (*n* = 6)^32^^,^^36–38^^,^^40^^,^^45^ and, in some studies, intervention protocols were not well described (*n* = 2).^36^^,^^40^

### Interventions

The duration of follow-up ranged from 8 weeks^42^ to 3 years,^39^ and the frequency of follow-up assessments varied greatly between studies, with some providing brief personalized support^44^ and others providing weekly follow-up^35^^,^^42^ throughout the study period.

Of the 15 unique studies included, 13 interventions were delivered with at least 1 face-to-face component.^32^^,^^34^^,^^36–46^ Seven included a combination of methods of delivery,^34^^,^^37^^,^^39^^,^^41–44^ while 2 interventions were primarily technology-based, delivered via telephone^35^ or app.^33^ In 2 studies, the majority of meals for the intervention period were provided to participants, with 1 study providing participants with easy-to-prepare meal boxes^43^ and another providing participants with 2 main meals per day.^33^ In another study, credit was provided to study participants to purchase food needed for the intervention diet.^42^ Of the 15 unique studies included, 8 interventions were led by dietitians,^32^^,^^35–38^^,^^43^^,^^45^^,^^46^ 2 studies were led by nutritionists,^39^^,^^41^ and 1 study was conducted by a nurse practitioner and included possible referral to a nutritionist.^34^ In a further 3 studies, the interventions were delivered by researchers,^33^^,^^40^^,^^42^ 1 of which delivered material developed by dietitians,^42^ and 1 intervention was delivered by a healthcare practitioner.^44^

Across the 15 included studies, the basis of personalization of the interventions for 2 studies were demographic and cultural characteristics of participants, including use of literacy-sensitive nutrition education modules^42^ and meal plans tailored to the Chinese language and culture.^46^ Another 3 studies were personalized based on participants’ health goals and values,^35^^,^^37^ or motivation for change.^36^ One study was personalized based on analysis of 51 metabolome biomarkers and 35 different single nucleotide polymorphisms (SNPs) for each participant.^33^ This was used to assign participants to metabolic “clusters,” which were then used to tailor nutrition advice and personalize their meal plans.^33^ The remaining studies (*n* = 9) gave dietary advice personalized according to an individual’s existing dietary patterns, which were evaluated through differing approaches during screening, including 3-day food records,^32^^,^^33^ food-frequency questionnaires (FFQs),^34^^,^^35^^,^^39^^,^^46^ and 24-hour recalls.^36^^,^^41^^,^^44^

### Comparators

The comparison groups included control groups receiving no intervention in 3 studies^32^^,^^39^^,^^44^ and usual care or standard advice in 9 unique studies.^34–38^^,^^40^^,^^41^^,^^45^^,^^46^ In the 3 studies where food or food credit was provided to the intervention group, the control group received identical treatment without the personalization of their meals,^33^ or the addition of PN advice.^42^^,^^43^ Three included studies had more than 1 intervention arm; however, only the study arms relevant to the review criteria were extracted and included (Table S3).^39^^,^^43^^,^^44^

### Outcome measures

Three of the included studies investigated the effects of PN on risk estimates for CVD (including the Copenhagen Risk Score,^45^ FRS,^37^ and an FRS recalibrated for the Chinese population^47^). One study reported a significant between-group improvement in CVD risk score (*P* < .05) in favor of the control group.^45^

Nine of the 15 unique included studies examined BP using either 24-hour ambulatory BP measurements^35^ or a self-recorded^37^ or clinic-based measurement.^32^^,^^33^^,^^40^^,^^42–44^^,^^46^ Three studies reported significant between-group improvement in SBP and DBP (*P* < .004) in the intervention group.^35^^,^^40^^,^^43^ Two were dietitian-administered MNT interventions,^35^^,^^43^ while the other was led by research nutritionists and focused on DASH diet principles.^40^ Notably, in all 3 studies, the experimental group had much more frequent and intensive intervention exposure than the control group—for example, a minimum of 4 appointments/meetings with interventionists in intervention arms compared with standard advice provided once-off at baseline in the control groups.

Thirteen included studies evaluated changes in blood lipid profiles.^32–34^^,^^36–39^^,^^41–46^ Of these, 9 studies^32–34^^,^^36^^,^^38^^,^^39^^,^^42–44^ were measured using fasting blood or plasma samples, while the other 4 did not provide details on laboratory methods used.^37^^,^^41^^,^^45^^,^^46^ Two studies resulted in significant between-group improvements in plasma lipid measurements in the intervention groups, including TC,^36^^,^^38^ LDL-C,^36^^,^^38^ high-density-lipoprotein cholesterol (HDL-C),^36^ and TG,^36^ compared with the controls for at least 1 follow-up. Notably, both study interventions were delivered by dietitians. In the study by Delahanty et al,^36^ significant improvements were observed in LDL-C, TC, TG, and HDL-C at 3-month follow-ups, although only TC remained significant at the end of the 6-month intervention. One study observed statistically significant between-group improvements in HDL-C in favor of the control group at follow-up.^45^ However, notably, during the study period, the control group had a significantly larger increase in the use of lipid-lowering medication than the intervention group (*P* = .004).

Fourteen studies reported on anthropometric outcomes,^32–37^^,^^39–46^ of which 11 reported on weight,^32–37^^,^^40^^,^^42–45^ 7 reported on BMI,^32^^,^^33^^,^^39–41^^,^^45^^,^^46^ and 6 reported on waist circumference change.^32^^,^^33^^,^^35^^,^^40^^,^^43^^,^^45^ As detailed in Table S3, 7 studies found statistically significant between-group differences in weight,^34^^,^^36^^,^^37^^,^^40^^,^^45^ BMI,^39^^,^^40^^,^^45^ and/or waist circumference^40^^,^^43^ in favor of the intervention groups. The 4 remaining studies did not result in any significant between-group differences in anthropometric measurements.^35^^,^^41^^,^^42^^,^^44^

Among the included studies, 10 investigated dietary intake outcomes. The most common dietary intake assessment method was FFQs,^34^^,^^35^^,^^39^^,^^46^ followed by 3-day food records,^32^^,^^33^ and 24-hour recalls.^36^^,^^44^ Of the 10 studies examining dietary outcomes, 6 measured intake of nutrients, including total energy,^32^^,^^33^^,^^35^^,^^36^^,^^39^^,^^44^ total fat,^32^^,^^33^^,^^36^^,^^39^^,^^42^^,^^44^ saturated fatty acid (SFA),^33^^,^^36^^,^^39^^,^^44^ monounsaturated fatty acid (MUFA),^33^^,^^36^^,^^39^^,^^44^ polyunsaturated fatty acid (PUFA),^33^^,^^36^^,^^39^^,^^44^ omega-3 and omega-6 fatty acids, cholesterol,^32^^,^^36^^,^^39^^,^^42^ protein,^32^^,^^33^^,^^39^ carbohydrate,^32^^,^^33^^,^^39^ fiber,^32^^,^^33^^,^^36^^,^^39^^,^^42^^,^^44^ vitamin C,^42^ vitamin B_6_,^32^ folate,^32^ potassium,^42^ sodium,^35^ and magnesium.^42^ Another 3 studies examined intake of food groups, 2 examining fruit and vegetable intake only^37^^,^^42^ and 1 examining a variety of food groups including fruits, vegetables, grains, dairy, meats, nuts, legumes and seeds, fats and oils, and sweets.^46^ Two studies measured intake using either a Healthy Eating Index (HEI) score^35^ or Dietary Risk Assessment (DRA) score.^34^

Of the 6 studies measuring nutrient intake, 5 found statistically significant between-group improvements in nutrient intake.^33^^,^^36^^,^^39^^,^^42^^,^^44^ This included decreases in total energy,^36^ total fat,^36^^,^^39^ SFA,^36^^,^^39^ MUFA,^36^^,^^39^ and cholesterol intake,^36^ and increases in intake of carbohydrate,^39^ fiber,^33^^,^^36^^,^^39^^,^^42^ PUFA,^39^^,^^44^ n-3 fatty acids,^39^ magnesium,^42^ potassium,^42^ and/or vitamin C^42^ in the intervention groups.

All 3 studies examining the intake of different food groups reported statistically significant between-group improvements for at least 1 dietary outcome, including increased intake of vegetables,^37^^,^^42^^,^^46^ fruit,^37^^,^^42^ and low-fat dairy foods.^46^ Of the 2 studies using dietary index scores to measure diet quality, 1 study showed no statistically significant changes in intake,^35^ while the other observed significant between-group improvements in the diet quality score.^34^

### Meta-analysis of Outcome Measures

Of the 15 included studies, 12 included dietary counseling interventions and were therefore deemed eligible for inclusion in the meta-analysis.^32^^,^^35–43^^,^^45^^,^^46^ Meta-analysis was performed for SBP, DBP, TC, TG, LDL-C, HDL-C, weight, waist circumference, and BMI. The CVD risk scores and dietary intake were not included due to the heterogeneity across studies in assessment tools used to record these outcomes.

**Figure 2.**
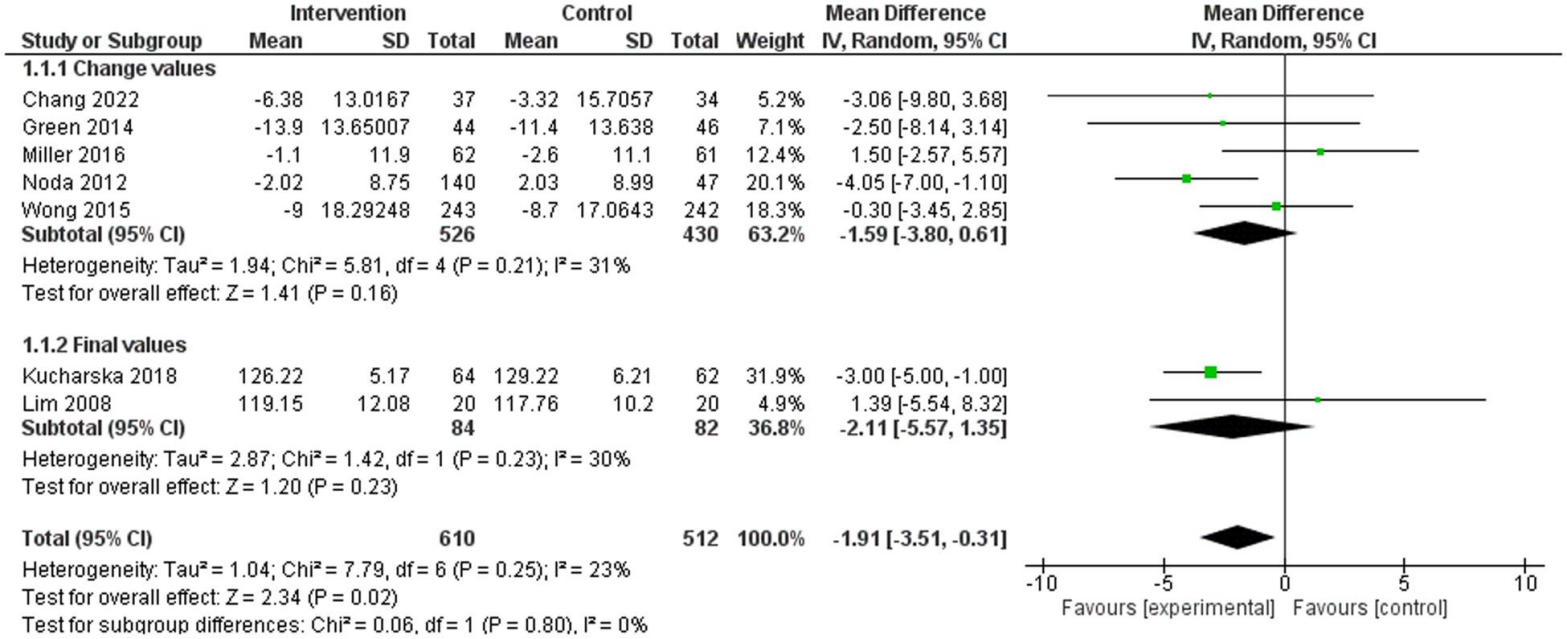
Meta-analysis Results for Systolic Blood Pressure (SBP). Abbreviation: IV, inverse variance

**Figure 3.**
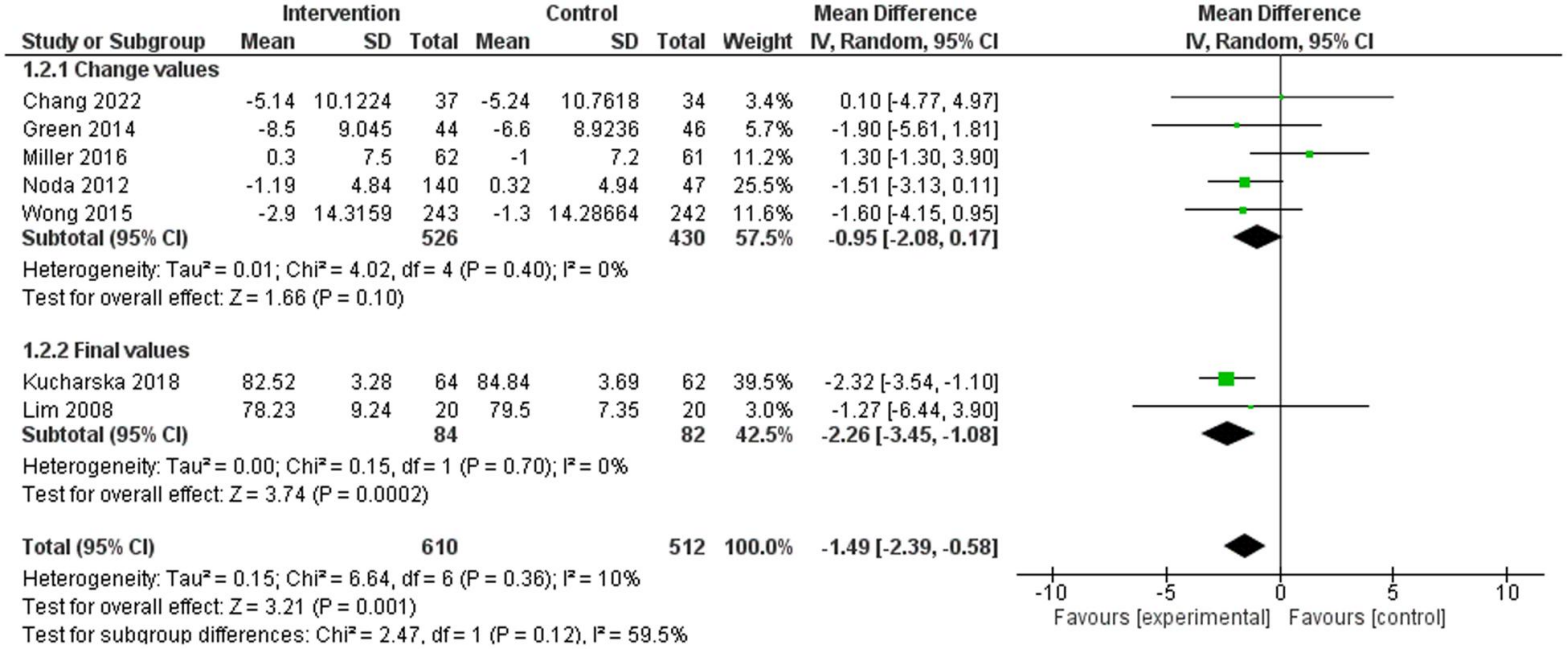
Meta-analysis Results for Diastolic Blood Pressure (DBP). Abbreviation: IV, inverse variance

Seven included studies reported on BP,^32^^,^^35^^,^^37^^,^^40^^,^^42^^,^^43^^,^^46^ with results indicating a significant reduction in both SBP (Figure 2) and DBP (Figure 3) in favor of dietary counseling groups. Ten studies in the meta-analysis reported on at least 1 blood lipid outcome.^32^^,^^36–39^^,^^41–43^^,^^45^^,^^46^ No significant differences were identified for TC, LDL-C, and HDL-C (Figures S1–S3). Significant difference in favor of the intervention group was observed for TG (weighted MD [WMD]: −0.15; 95% CI: −0.27, −0.02 mmol/L) favoring the intervention group (Figure S4). Eleven studies included in the meta-analysis reported on at least 1 anthropometric outcome.^32^^,^^35–37^^,^^39–43^^,^^45^^,^^46^ Statistically significant differences were observed in weight (WMD: −1.49; 95% CI: −2.62, −0.36 kg) and BMI (WMD: −0.59; 95% CI: −1.04, −0.14 kg/m^2^) favoring the intervention group (Figures S5 and S6), while no statistically significant differences were observed in waist circumference (Figure S7).

### Meta-analysis Subgroup Analysis Dietitian-Only Interventions

**Figure 4.**
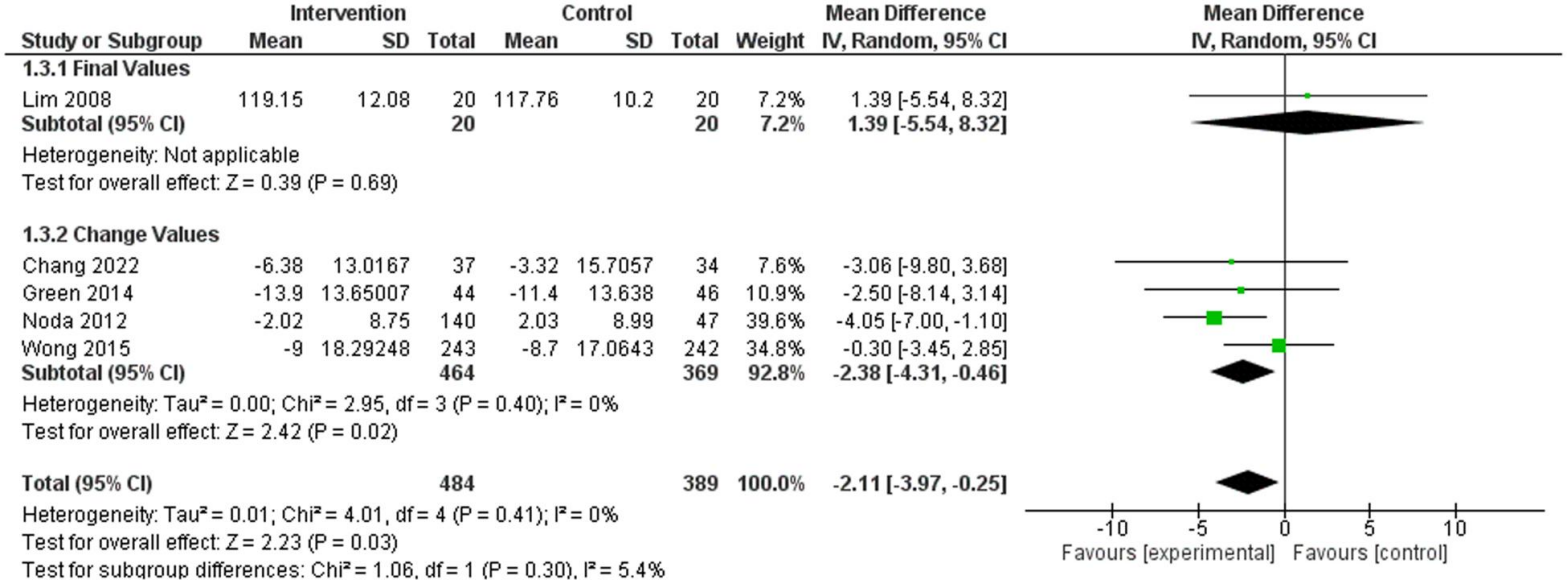
Meta-analysis Results for Systolic Blood Pressure (SBP) Dietitian Subgroup Analysis. Abbreviation: IV, inverse variance

**Figure 5.**
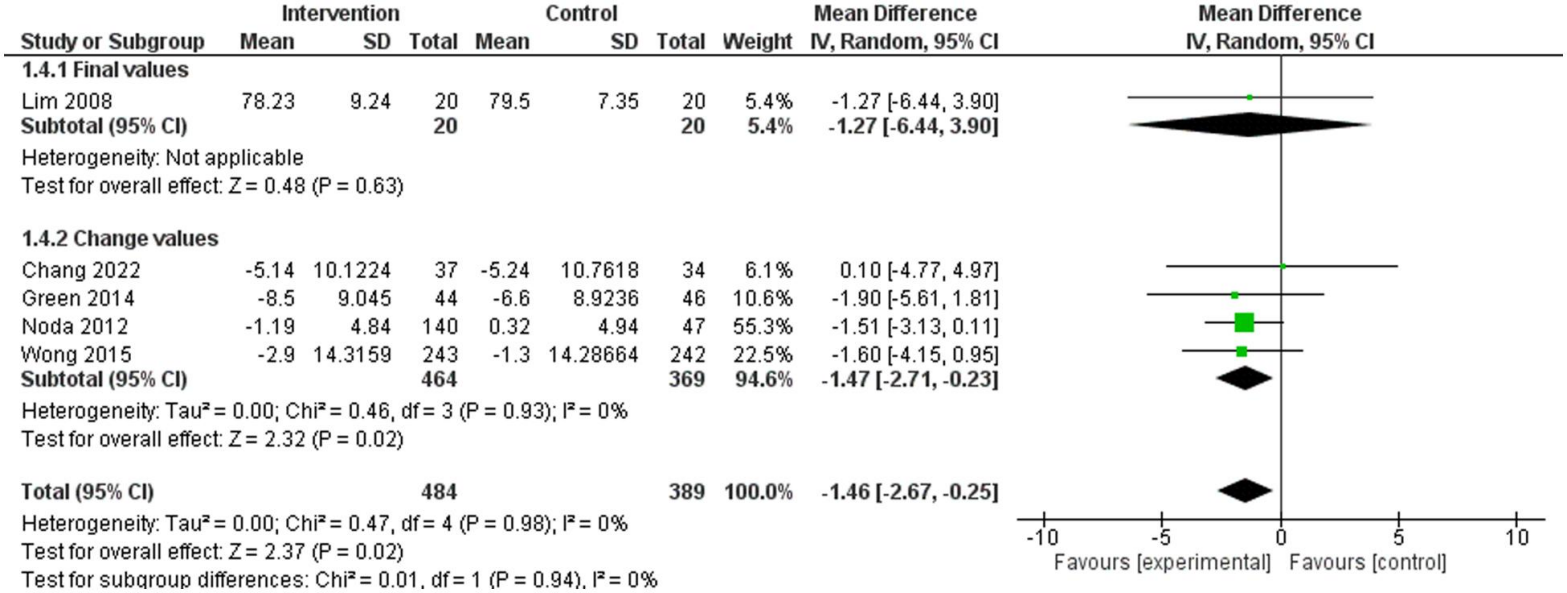
Meta-analysis Results for Diastolic Blood Pressure (DBP) Dietitian Subgroup Analysis. Abbreviation: IV, inverse variance

**Figure 6.**
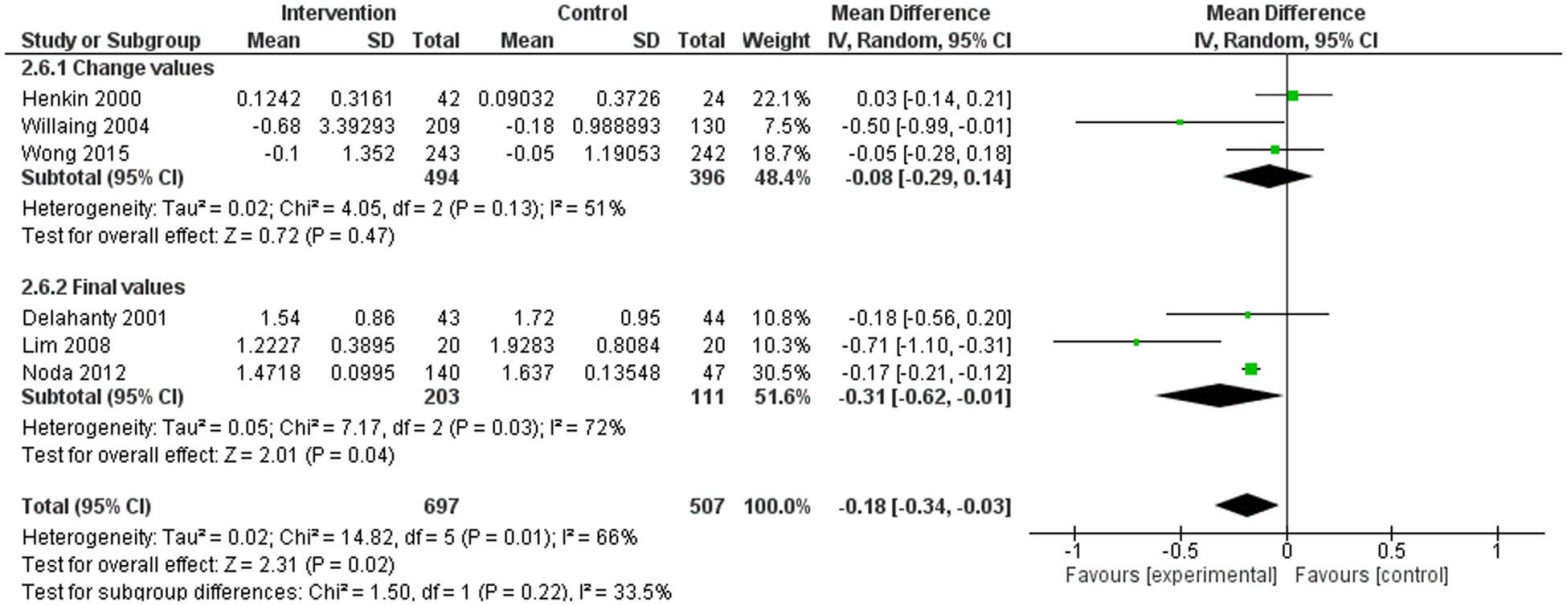
Meta-analysis Results for Triglycerides (TG) Dietitian Subgroup Analysis. Abbreviation: IV, inverse variance

A subgroup analysis was performed examining the same outcomes (SBP, DBP, TC, TG, LDL-C, HDL-C, weight, waist circumference, and BMI) in dietitian-led studies only.^32^^,^^35–38^^,^^43^^,^^45^^,^^46^ There were statistically significant differences in SBP (WMD: −2.11; 95% CI: −3.97, −0.25 mmHg), DBP (WMD: −1.46; 95% CI: −2.67, −0.25 mmHg), and TG (WMD: −0.18; 95% CI: −0.34, −0.03 units) favoring the dietitian-led intervention groups (Figures  4–6). Further subgroup analysis was undertaken for TC, LDL-C, HDL-C, weight, BMI, or waist circumference (Figures S8–S13). Only weight (Figure S11) was significant, favoring the dietitian-led intervention groups.

## DISCUSSION

The current systematic review and meta-analysis synthesized the effectiveness of PN on CVD risk factors and dietary intake in individuals at elevated CVD risk. Evidence from the included RCTs suggests that PN interventions are more effective at improving BP and dietary intake than usual care or minimal care comparators. Findings regarding CVD risk scores, plasma lipids, and anthropometric measures were less consistent. This may be explained, in part, by the heterogeneity across studies in terms of the methodology, study length, frequency of engagement with intervention components, qualifications of interventionists responsible for delivery, and differences between control and comparator groups across studies.

Personalized nutrition interventions are currently unclearly defined, but broadly incorporate analysis of current behaviors, genetic and phenotypic characteristics, and biological responses to diet.^48^ Additionally, the current review widened the scope of this definition by including MNT by dietitians due to the similarities in methodological approaches with other PN interventions. The included studies were primarily personalized based on behavior and lifestyle characteristics, while only 1 study used genetic and metabolomic information to personalize the dietary intervention.^33^ Hence, the evidence synthesis is not able to produce consistent benefits due to CVD-specific PN. There was also large variation in the application of personalization within the nutrition interventions, similar to findings from reviews by Robertson et al^18^ and Jinnette et al^19^ of PN on dietary intake, which reported significant heterogeneity across the included studies that precluded meta-analysis. A more comprehensive examination of the basis of personalization is needed in future studies to better synthesize the effectiveness of PN in the prevention and management of CVD.

Overall, evidence from the present review supported the benefit of PN interventions on BP outcomes. Meta-analysis results found statistically significant decreases in both SBP and DBP in favor of the intervention groups. Further, the sensitivity analysis found further change in favor of dietitian-led intervention groups, which is consistent with other meta-analyses that showed that dietitian-led MNT reduced BP outcomes.^10^ It is important to note, however, that the meta-analysis in the current study was limited to dietary counseling studies only and was not necessarily representative of all PN interventions.

The current review found mixed results for the effect of PN interventions on plasma lipid levels, with the meta-analysis results only showing statistically significant reductions in TG within PN dietary intervention groups and no significant changes in TC, LDL-C, or HDL-C. There were 2 studies that found statistically significant between-group improvements in plasma lipids,^36^^,^^38^ in which the interventions were delivered by dietitians; however, this finding was not verified in the meta-analysis in the subgroup analysis of dietitian-led studies. Findings from the meta-analysis were not consistent with findings from systematic reviews by Mohr et al^12^ and Ross et al,^13^ who reported MNT by dietitians to be effective in improving plasma lipid measurements. In the studies within the present review that found significant differences in at least 1 plasma lipid outcome,^36^^,^^38^ dietary counseling studies focused specifically on the treatment of dyslipidemia and utilized cardioprotective diets, such as a reduction in total SFA intake^44^ or the National Cholesterol Education Program step II diet, which has stringent targets for total fat, saturated fat, and cholesterol intake.^36^^,^^38^ It is also important to note that a number of the other included studies investigating plasma lipids had intervention periods as short as 8 weeks. This may limit the magnitude of effect observed, as prior research suggests that lifestyle interventions can take 3 months to detect significant changes in blood lipids.^49^ Therefore, the duration of the intervention is an important consideration.

Anthropometry outcomes were variable. Some significant improvements in weight, BMI, and/or waist circumference were observed favoring the intervention groups. Importantly, not all included studies recommended or focused on weight loss/caloric restriction. Of the 7 studies that did recommend energy restriction,^33^^,^^37^^,^^39–41^^,^^43^^,^^45^ the majority reported statistically significant improvements in anthropometric outcomes.^37^^,^^39^^,^^40^^,^^45^ This was confirmed by the meta-analysis, within which 6 of the 7 studies recommending energy restriction were included and found statistically significant changes in weight and BMI favoring the dietary counseling intervention groups.

Significant between-group differences in dietary intake were observed in the present study, including intakes of total energy and various nutrients and intake of specific food groups. While these findings supported the potential benefits of PN interventions on dietary outcomes, this may equally be reflective of the inclusion of primarily dietitian-led intervention studies, which are known to improve dietary intake outcomes.^50^ Due to the heterogeneity in the reported dietary outcomes, this could not be meta-analyzed; however, anecdotally more promising outcomes for the benefits of PN on dietary intake were shown in interventions based on current eating habits, behaviors, and phenotypic characteristics than based on genetic and metabolomic information. This is consistent with the findings in the systematic review by Jinnette et al,^19^ which similarly found communication of genetics-based risk to have little or no impact on improving dietary intake. However, more research is needed to strengthen the evidence base, as both reviews included very few studies investigating genetic and/or metabolomic nutrition personalization. Despite the potential success of behavioral-level PN on dietary outcomes, there is a lack of consistency in study methodology, data-collection methods, and reporting, as well as a lack of studies reporting on long-term maintenance of outcomes. In the 2 included studies that included follow-ups post-intervention, no intervention improvements were maintained. This supports the recommendation by Biesiekierski et al^48^ to introduce specific behavior-change models within PN interventions, in order to promote lasting behavioral change. Future PN studies could potentially include individual stages of change^51^ or personal activation level^52^ in the design of PN interventions.

### Strengths and Limitations

There were numerous strengths of the present review, including, first, the inclusion of RCTs only and the conduction of a meta-analysis. As well as this, the included studies were all rated high-quality during quality assessment. Another strength was the use of a broad definition of PN that included MNT, which is often viewed separately, despite encompassing the same criteria as PN. This is important in terms of capturing interventions delivered by dietitians who have the specific nutrition expertise and training in regard to both nutrition science and behavior change counseling.

The heterogeneity of studies was a primary limitation of the current review. The designs of PN interventions, including intervention length, frequency of follow-up, methods of nutrition personalization, and methods of measuring outcomes, were highly heterogeneous. Additionally, a number of studies failed to adequately document intervention protocols, process fidelity, participant compliance, and descriptions of comparator group treatment. It was therefore difficult to compare some results between studies and this introduces limitations to reproducing and/or building upon these interventions.

For studies included in the meta-analysis, several did not report mean differences of change and SDs. As per the Cochrane guidelines, when data were unavailable, final post-intervention mean and SD values for each intervention and comparator group were used instead.^29^ This introduces a significant issue due to the baseline differences between the intervention and control groups in some studies, which may potentially influence the interpretation of the true intervention effect. To address this, we sought additional data from authors, specifically values of change from baseline to post-treatment for intervention and comparator groups; however, no authors were able to provide this information. As such, caution should be used when interpreting and generalizing between-group results, as baseline discrepancies can confound or exaggerate outcome effects. Additional sensitivity analyses were conducted as part of the meta-analysis, which involved excluding each article sequentially to explore the impact of each individual study on the overall pooled effect. A subgroup analysis separating results that reported mean change from baseline from post-intervention values was also performed to confirm how these differences may skew results.

Another limitation was the reporting of dietary outcomes, as there was significant variability in the methods of dietary intake data collection and the type of information collected. Additionally, dietary information was self-reported in all included studies, introducing potential recall and reporting bias. Future studies could consider using objective biomarkers of dietary intake alongside self-reported intake to support findings.

### Recommendations for Future Research and Practice

Longer-term interventions would enable a stronger evidence base for the effects of PN in CVD prevention and management, by examining the magnitude of changes in outcome measures that may take longer to detect, such as blood lipids and anthropometry.^49^ Follow-up periods of 12 months or longer would more adequately capture participants’ adherence to dietary advice long term, as well as its long-term impacts on cardiometabolic and anthropometric outcomes, as seen in the systematic review by Dombrowski et al.^53^ Longer intervention periods would also provide interventionists with greater opportunities to review and reinforce dietary advice to promote habit-forming for lasting behavior change.^54^

The use of more consistent means of measuring biometric outcomes would also enable a stronger evidence base in future studies. For example, acknowledging the limitations of using one-off clinical measurements for BP, consistent use of a 24-hour BP measurement in future studies would provide more reliable and comparable results.^55^ It is also recommended that future PN interventions use more rigorous dietary intake data-collection instruments and standardize diet-related outcomes to improve the comparability of dietary intake between studies. One approach to improving estimates of dietary intake data is using combined information from different self-report instruments.^56^ In the study by Freedman et al^56^ combining an FFQ with multiple 24-hour recalls was shown to modestly improve accuracy of estimates of individual intakes. Additionally, future studies could consider using objective biomarkers of dietary intake along with self-reported intake to support findings—for example, red blood cell membrane fatty acids and plasma carotenoids. Finally, future studies could incorporate use of validated behavior-change models and techniques to guide the intervention delivery in future PN studies.^57^

## Conclusion

While the present review found some promising findings for the effectiveness of PN interventions on CVD risk factors and dietary intake in people at elevated CVD risk, the current body of evidence is too limited to provide definitive results outside of personalization based on dietary counseling interventions. A larger evidence base of PN trials with more well-designed interventions is needed to better understand the effectiveness of CVD-specific PN. Hence, it is recommended that future studies more rigorously document study protocols and intervention details in order to strengthen research in the PN field.

## Supplementary Material

nuae149_Supplementary_Data

## Data Availability

All data are available from the included manuscripts.
