# Supplementary material for: Do Personalized Nutrition Interventions Improve Dietary Intake and Risk Factors in Adults With Elevated Cardiovascular Disease Risk Factors? A Systematic Review and Meta-analysis of Randomized Controlled Trials
Source: Nutr Rev. 2024 Oct 17;83(7):e1709–21. doi: 10.1093/nutrit/nuae149 (PMC12166176; doi:10.1093/nutrit/nuae149)

**Supplementary data**

**Title: Do precision and personalised nutrition interventions improve dietary intake and risk factors in adults with elevated cardiovascular disease (CVD) risk factors? A systematic review and meta-analysis of randomised controlled trials**

**Authors name:**

Victoria Cross, ^1^ BNut&Diet.

Jordan Stanford, ^1,2^ APD, PhD. <https://orcid.org/0000-0003-3541-8960>

Maria Gomez-Martin, ^1,2^ PhD. <https://orcid.org/0000-0001-7300-7471>

Seaton Robertson, ^1^ BNut&Diet.

Clare E Collins,^1,2^ FDA, PhD. <https://orcid.org/0000-0003-3298-756X>

Erin D Clarke,^1,2^ APD, PhD. <https://orcid.org/0000-0001-8250-5990>

**Author Affiliations:**

1. School of Health Sciences, College of Health, Medicine and Wellbeing, The University of Newcastle, Callaghan, NSW, 2308 Australia
2. Food and Nutrition Research Program, Hunter Medical Research Institute, New Lambton Heights, NSW, 2305 Australia

**Corresponding author:**Dr Erin Clarke

Advanced Technology Centre, Room 310, The University of Newcastle, University Drive, Callaghan, NSW 2308, Australia

Email: [erin.clarke@newcastle.edu.au](mailto:erin.clarke@newcastle.edu.au)

Tel.: +61 2 4921 8834

**Key words:** Cardiovascular disease, personalised nutrition, medical nutrition therapy, risk factors, diet.

**Supplementary Table 1** PRISMA Reporting Guidelines Checklist

| **Section and Topic** | **Item #** | **Checklist item** | **Location where item is reported** |
| --- | --- | --- | --- |
| **TITLE** | | |  |
| Title | 1 | Identify the report as a systematic review. | Page 1 |
| **ABSTRACT** | | |  |
| Abstract | 2 | See the PRISMA 2020 for Abstracts checklist. | Page 3 |
| **INTRODUCTION** | | |  |
| Rationale | 3 | Describe the rationale for the review in the context of existing knowledge. | Pages 5-6 |
| Objectives | 4 | Provide an explicit statement of the objective(s) or question(s) the review addresses. | Page 6 |
| **METHODS** | | |  |
| Eligibility criteria | 5 | Specify the inclusion and exclusion criteria for the review and how studies were grouped for the syntheses. | Page 7 |
| Information sources | 6 | Specify all databases, registers, websites, organisations, reference lists and other sources searched or consulted to identify studies. Specify the date when each source was last searched or consulted. | Page 8 |
| Search strategy | 7 | Present the full search strategies for all databases, registers and websites, including any filters and limits used. | Pages 24-33 |
| Selection process | 8 | Specify the methods used to decide whether a study met the inclusion criteria of the review, including how many reviewers screened each record and each report retrieved, whether they worked independently, and if applicable, details of automation tools used in the process. | Page 8 |
| Data collection process | 9 | Specify the methods used to collect data from reports, including how many reviewers collected data from each report, whether they worked independently, any processes for obtaining or confirming data from study investigators, and if applicable, details of automation tools used in the process. | Page 8-9 |
| Data items | 10a | List and define all outcomes for which data were sought. Specify whether all results that were compatible with each outcome domain in each study were sought (e.g. for all measures, time points, analyses), and if not, the methods used to decide which results to collect. | Page 9 |
|  | 10b | List and define all other variables for which data were sought (e.g. participant and intervention characteristics, funding sources). Describe any assumptions made about any missing or unclear information. | Page 9 |
| Study risk of bias assessment | 11 | Specify the methods used to assess risk of bias in the included studies, including details of the tool(s) used, how many reviewers assessed each study and whether they worked independently, and if applicable, details of automation tools used in the process. | Page 9 |
| Effect measures | 12 | Specify for each outcome the effect measure(s) (e.g. risk ratio, mean difference) used in the synthesis or presentation of results. | Page 34-44 |
| Synthesis methods | 13a | Describe the processes used to decide which studies were eligible for each synthesis (e.g. tabulating the study intervention characteristics and comparing against the planned groups for each synthesis (item #5)). | Page 9 |
|  | 13b | Describe any methods required to prepare the data for presentation or synthesis, such as handling of missing summary statistics, or data conversions. | NA |
|  | 13c | Describe any methods used to tabulate or visually display results of individual studies and syntheses. | NA |
|  | 13d | Describe any methods used to synthesize results and provide a rationale for the choice(s). If meta-analysis was performed, describe the model(s), method(s) to identify the presence and extent of statistical heterogeneity, and software package(s) used. | NA |
|  | 13e | Describe any methods used to explore possible causes of heterogeneity among study results (e.g. subgroup analysis, meta-regression). | NA |
|  | 13f | Describe any sensitivity analyses conducted to assess robustness of the synthesized results. | NA |
| Reporting bias assessment | 14 | Describe any methods used to assess risk of bias due to missing results in a synthesis (arising from reporting biases). | Page 45 |
| Certainty assessment | 15 | Describe any methods used to assess certainty (or confidence) in the body of evidence for an outcome. | NA |
| **RESULTS** | | |  |
| Study selection | 16a | Describe the results of the search and selection process, from the number of records identified in the search to the number of studies included in the review, ideally using a flow diagram. | Page 10 |
|  | 16b | Cite studies that might appear to meet the inclusion criteria, but which were excluded, and explain why they were excluded. | Page 10 |
| Study characteristics | 17 | Cite each included study and present its characteristics. | Pages 11-13 |
| Risk of bias in studies | 18 | Present assessments of risk of bias for each included study. | Page 45 |
| Results of individual studies | 19 | For all outcomes, present, for each study: (a) summary statistics for each group (where appropriate) and (b) an effect estimate and its precision (e.g. confidence/credible interval), ideally using structured tables or plots. | Page 34-44 |
| Results of syntheses | 20a | For each synthesis, briefly summarise the characteristics and risk of bias among contributing studies. | Page 14-16 |
|  | 20b | Present results of all statistical syntheses conducted. If meta-analysis was done, present for each the summary estimate and its precision (e.g. confidence/credible interval) and measures of statistical heterogeneity. If comparing groups, describe the direction of the effect. | NA |
|  | 20c | Present results of all investigations of possible causes of heterogeneity among study results. | NA |
|  | 20d | Present results of all sensitivity analyses conducted to assess the robustness of the synthesized results. | Pages 14-16 |
| Reporting biases | 21 | Present assessments of risk of bias due to missing results (arising from reporting biases) for each synthesis assessed. | Page 45 |
| Certainty of evidence | 22 | Present assessments of certainty (or confidence) in the body of evidence for each outcome assessed. | NA |
| **DISCUSSION** | | |  |
| Discussion | 23a | Provide a general interpretation of the results in the context of other evidence. | Pages 16-21 |
|  | 23b | Discuss any limitations of the evidence included in the review. | Page 20 |
|  | 23c | Discuss any limitations of the review processes used. | Page 20 |
|  | 23d | Discuss implications of the results for practice, policy, and future research. | Pages 20-21 |
| **OTHER INFORMATION** | | |  |
| Registration and protocol | 24a | Provide registration information for the review, including register name and registration number, or state that the review was not registered. | Page 6 |
|  | 24b | Indicate where the review protocol can be accessed, or state that a protocol was not prepared. | Page 6 |
|  | 24c | Describe and explain any amendments to information provided at registration or in the protocol. | NA |
| Support | 25 | Describe sources of financial or non-financial support for the review, and the role of the funders or sponsors in the review. | Page 1 |
| Competing interests | 26 | Declare any competing interests of review authors. | Page 1 |
| Availability of data, code and other materials | 27 | Report which of the following are publicly available and where they can be found: template data collection forms; data extracted from included studies; data used for all analyses; analytic code; any other materials used in the review. | NA |

**Supplementary Table 2.** Search Strategy

1. Medline Search Strategy

Database(s): **Ovid MEDLINE(R)**1996 to May 05, 2023
Search Strategy:

| **#** | **Searches** | **Results** |
| --- | --- | --- |
| 1 | heart disease.mp. or Heart Diseases/ | 159203 |
| 2 | Cholesterol/ or cholesterol.mp. | 204570 |
| 3 | Hyperlipidemias/ or hyperlipid*.mp. | 34505 |
| 4 | Hypercholesterolemia/ or hypercholesterol*.mp. | 34252 |
| 5 | blood pressure.mp. or Blood Pressure/ | 277256 |
| 6 | Cardiovascular Diseases/ or cardiovascular health.mp. | 153895 |
| 7 | cardiovascular disease*.mp. | 256921 |
| 8 | 1 or 2 or 3 or 4 or 5 or 6 or 7 | 787768 |
| 9 | Diet/ or personalised diet*.mp. | 129259 |
| 10 | personalized diet*.mp. | 310 |
| 11 | Nutrigenomics/ or nutrigenomic*.mp. | 1424 |
| 12 | Nutrition Therapy/ or individualised nutrition*.mp. | 2809 |
| 13 | individualized nutrition*.mp. | 305 |
| 14 | gene.mp. or Genes/ | 2145750 |
| 15 | genes.mp. | 1115392 |
| 16 | DNA/ or DNA.mp. | 1327593 |
| 17 | genomic*.mp. | 317517 |
| 18 | Genotype/ or genotyp*.mp. | 379902 |
| 19 | medical nutrition therapy.mp. | 697 |
| 20 | Diet Therapy/ or diet therap*.mp. | 42335 |
| 21 | counsel*.mp. | 111138 |
| 22 | nutrition therapy.mp. | 4213 |
| 23 | 9 or 10 or 11 or 12 or 13 or 14 or 15 or 16 or 17 or 18 or 19 or 20 or 21 or 22 | 3534585 |
| 24 | Diet/ or diet*.mp. | 567103 |
| 25 | nutrition*.mp. or Nutrition Therapy/ | 298809 |
| 26 | 24 or 25 | 743230 |
| 27 | effect*.mp. | 7251474 |
| 28 | outcome*.mp. | 2654238 |
| 29 | change*.mp. | 2362124 |
| 30 | modif*.mp. | 943517 |
| 31 | 27 or 28 or 29 or 30 | 10071534 |
| 32 | recommendation*.mp. | 260824 |
| 33 | advice*.mp. | 44054 |
| 34 | information.mp. | 1162997 |
| 35 | program*.mp. | 867474 |
| 36 | counsel*.mp. | 111138 |
| 37 | intervention*.mp. | 1080829 |
| 38 | 32 or 33 or 34 or 35 or 36 or 37 | 3022886 |
| 39 | RCT.mp. | 27006 |
| 40 | randomised controlled trial.mp. | 26778 |
| 41 | randomized controlled trial.mp. | 522949 |
| 42 | randomised intervention.mp. | 272 |
| 43 | randomized intervention.mp. | 1131 |
| 44 | 39 or 40 or 41 or 42 or 43 | 544718 |
| 45 | 8 and 23 and 31 and 38 and 44 | 3569 |
| 46 | limit 45 to (english language and humans and randomized controlled trial and last 23 years) | 3072 |

1. Embase Search Strategy

Database(s): **Ovid Embase**1947 to present

| **#** | **Searches** | **Results** |
| --- | --- | --- |
| 1 | heart disease.mp. or Heart Diseases/ | 502436 |
| 2 | Cholesterol/ or cholesterol.mp. | 543788 |
| 3 | Hyperlipidemias/ or hyperlipid*.mp. | 113877 |
| 4 | Hypercholesterolemia/ or hypercholesterol.mp. | 75374 |
| 5 | blood pressure.mp. or Blood Pressure/ | 783018 |
| 6 | Cardiovascular Diseases/ or cardiovascular health.mp. | 128305 |
| 7 | cardiovascular disease.mp. or Cardiovascular Diseases/ | 476375 |
| 8 | cardiovascular diseases.mp. or Cardiovascular Diseases/ | 201770 |
| 9 | 1 or 2 or 3 or 4 or 5 or 6 or 7 or 8 | 2089114 |
| 10 | Diet/ or personalised diet*.mp. | 298391 |
| 11 | personalized diet.mp. | 241 |
| 12 | nutrigenomic.mp. or Nutrigenomics/ | 1948 |
| 13 | Diet/ or Nutrition Therapy/ or individualised nutrition.mp. | 362386 |
| 14 | individualized nutrition.mp. | 239 |
| 15 | gene.mp. or Genes/ | 4438491 |
| 16 | genes.mp. or Genes/ | 1818505 |
| 17 | DNA/ or DNA.mp. | 2363831 |
| 18 | genomic*.mp. | 569486 |
| 19 | Genotype/ or genotyp*.mp. | 640371 |
| 20 | medical nutrition therapy.mp. | 1237 |
| 21 | nutrition therapy.mp. or Nutrition Therapy/ | 69833 |
| 22 | 10 or 11 or 12 or 13 or 14 or 15 or 16 or 17 or 18 or 19 or 20 or 21 | 6316606 |
| 23 | diet*.mp. or Diet/ | 1255833 |
| 24 | nutrition*.mp. | 658847 |
| 25 | 23 or 24 | 1668630 |
| 26 | effect*.mp. | 12335666 |
| 27 | outcome*.mp. | 4456203 |
| 28 | change*.mp. | 4903927 |
| 29 | impact*.mp. | 2244008 |
| 30 | modif*.mp. | 1821226 |
| 31 | 26 or 27 or 28 or 29 or 30 | 19124807 |
| 32 | recommendation*.mp. | 485182 |
| 33 | advice*.mp. | 93883 |
| 34 | information.mp. | 2437143 |
| 35 | program*.mp. | 1816689 |
| 36 | counsel*.mp. | 278725 |
| 37 | intervention*.mp. | 1956060 |
| 38 | 32 or 33 or 34 or 35 or 36 or 37 | 6021627 |
| 39 | 9 and 22 and 31 and 38 | 33524 |
| 40 | limit 39 to (english language and humans and randomized controlled trial) | 3520 |

1. PubMed Search Strategy

| **#** | **Searches** | **Results** |
| --- | --- | --- |
| 1 | Search: ("heart disease" or "cholesterol" or "hyperlipid*" or "hypercholesterol*" or "blood pressure" or "cardiovascular health" or "cardiovascular disease" or "cardiovascular diseases"[mesh]) AND ("personalised diet*" or "personalized diet" or "nutrigenomic" or "individualised nutrition*" or "individualized nutrition*" or "gene" or "genes" or "dna" or "genomic*" or "genotype" or "medical nutrition therapy" or "nutrition therapy" [mesh] or "diet therapy" or "counsel*") AND (diet* or nutrition*) AND (effect* or outcome* or change* or impact* or modif*) and (recommendation* or advice* or information or program* or counsel* or intervention*) AND ("RCT" or "randomised controlled trial" or "randomized controlled trial" or "randomised intervention" or "randomized intervention") - Saved search Filters: Full text, Randomized Controlled Trial, Humans, English, from 2000/1/1 - 3000/12/12 Sort by: Most Recent  (("heart disease"[All Fields] OR "cholesterol"[All Fields] OR "hyperlipid*"[All Fields] OR "hypercholesterol*"[All Fields] OR "blood pressure"[All Fields] OR "cardiovascular health"[All Fields] OR "cardiovascular disease"[All Fields] OR "cardiovascular diseases"[MeSH Terms]) AND ("personalised diet*"[All Fields] OR "personalized diet"[All Fields] OR "nutrigenomic"[All Fields] OR "individualised nutrition*"[All Fields] OR "individualized nutrition*"[All Fields] OR "gene"[All Fields] OR "genes"[All Fields] OR "dna"[All Fields] OR "genomic*"[All Fields] OR "genotype"[All Fields] OR "medical nutrition therapy"[All Fields] OR "nutrition therapy"[MeSH Terms] OR "diet therapy"[All Fields] OR "counsel*"[All Fields]) AND ("diet*"[All Fields] OR "nutrition*"[All Fields]) AND ("effect*"[All Fields] OR "outcome*"[All Fields] OR "change*"[All Fields] OR "impact*"[All Fields] OR "modif*"[All Fields]) AND ("recommendation*"[All Fields] OR "advice*"[All Fields] OR ("inform"[All Fields] OR "informal"[All Fields] OR "informality"[All Fields] OR "informally"[All Fields] OR "informant"[All Fields] OR "informant s"[All Fields] OR "informants"[All Fields] OR "information"[All Fields] OR "information s"[All Fields] OR "informational"[All Fields] OR "informations"[All Fields] OR "informative"[All Fields] OR "informatively"[All Fields] OR "informativeness"[All Fields] OR "informativity"[All Fields] OR "informed"[All Fields] OR "informer"[All Fields] OR "informers"[All Fields] OR "informing"[All Fields] OR "informs"[All Fields]) OR "program*"[All Fields] OR "counsel*"[All Fields] OR "intervention*"[All Fields]) AND ("RCT"[All Fields] OR "randomised controlled trial"[All Fields] OR "randomized controlled trial"[All Fields] OR "randomised intervention"[All Fields] OR "randomized intervention"[All Fields])) AND ((randomizedcontrolledtrial[Filter]) AND (fft[Filter]) AND (humans[Filter]) AND (2000/1/1:3000/12/12[pdat]) AND (english[Filter])) | 2629 |

d) Cochrane Library Search Strategy

| ID | Query | Search Hits |
| --- | --- | --- |
| #1 | ("heart disease" OR cholesterol OR hyperlipid* OR hypercholesterol OR "blood pressure" OR "cardiovascular health" OR "cardiovascular disease" OR [mh "cardiovascular diseases"]) AND (("personalised" NEXT diet*) OR "personalized diet" OR nutrigenomic OR ("individualised" NEXT nutrition*) OR ("individualized" NEXT nutrition*) OR gene OR genes OR dna OR genomic* OR genotype OR "medical nutrition therapy" OR [mh "nutrition therapy"] OR "diet therapy" OR counsel* ) AND (diet* OR nutrition* ) AND (effect* OR outcome* OR change* OR impact* OR modif* ) AND (recommendation* OR advice* OR information OR program* OR counsel* OR intervention* ) AND (RCT OR "randomised controlled trial" OR "randomized controlled trial" OR "randomised intervention" OR "randomized intervention" ) with Publication Year from 2000 to 2023, in Trials | 4081 |

e) CINAHL Search Strategy

| **#** | **Query** | **Limiters/Expanders** | **Last Run Via** | **Results** |
| --- | --- | --- | --- | --- |
| S1 | ( "heart disease" or "cholesterol" or "hyperlipid*" or "hypercholesterol*" or "blood pressure" or "cardiovascular health" or "cardiovascular disease" or "cardiovascular diseases" ) AND ( "personalised diet*" or "personalized diet" or "nutrigenomic" or "individualised nutrition*" or "individualized nutrition*" or "gene" or "genes" or "dna" or "genomic*" or "genotype" or "medical nutrition therapy" or "nutrition therapy" or “diet therapy” or “counsel*” ) AND ( diet* or nutrition* ) AND ( effect* or outcome* or change* or impact* or modif*) and (recommendation* or advice* or information or program* or counsel* or intervention* ) AND ( “RCT” or “randomised controlled trial” or “randomized controlled trial” or “randomised intervention” or **“**randomized intervention” ) | Limiters - Published Date: 20000101-20231231; English Language; Human Expanders - Apply equivalent subjects Search modes - Boolean/Phrase | Interface - EBSCOhost Research Databases Search Screen - Advanced Search Database - CINAHL Complete | 276 |

f) Scopus Search Strategy

| **#** | **Searches** | **Results** |
| --- | --- | --- |
| 1 | (“heart disease” OR “cholesterol” OR “hyperlipid*” OR “hypercholesterol*” OR “blood pressure” OR “cardiovascular health” OR “cardiovascular disease” OR “cardiovascular diseases” [mesh]) AND (“personalised diet*” OR “personalized diet” OR “nutrigenomic” OR “individualised nutrition*” OR “individualized nutrition*” OR “gene” OR “genes” OR “dna” OR “genomic*” OR “genotype” OR “medical nutrition therapy” OR “nutrition therapy” [mesh] OR “diet therapy” OR “counsel*”) AND (diet* OR nutrition*) AND (effect* OR outcome* OR change* OR impact* OR modif*) AND (recommendation* OR advice* OR information OR program* OR counsel* OR intervention*) AND (EXCLUDE (PUBYEAR 1998) OR EXCLUDE (PUBYEAR, 1997) OR EXCLUDE (PUBYEAR 1996) OR EXCLUDE (PUBYEAR, 1992)) AND LIMIT-TO (LANGUAGE, “English”)) | 1264 |

**Supplementary Table 3** Results of included studies

| First author, year, country | Primary risk | Participant Characteristics | RCT design, and study duration | Outcomes measured | Intervention prescribed by | Intervention group(s) conditions | Comparison group(s) conditions | CVD Risk Outcomes | Anthropometry Outcomes | Dietary Outcomes | Author’s conclusion |
| --- | --- | --- | --- | --- | --- | --- | --- | --- | --- | --- | --- |
| Aldubayan et al., 2022, Denmark | Overweight/  obesity | Baseline n=100, follow-up n= 82 *Intervention*: mean age: 45.50 ±11.80 yrs,75% F. *Control*: mean age 45.00 ± 11.40 yrs,63% F | Parallel, 10 weeks | Blood lipids (TC, LDL-C, HDL-C, TG), blood pressure (SBP, DBP), anthropometry (weight, BMI, waist circumference (WC), fat and lean body mass, visceral adipose tissue, total body fat), dietary intake (total energy, fat %E, SFA, PUFA, MUFA, protein %E carbohydrate %E, fibre, added sugar) | Intervention & Comparators: researchers | Provided personalised meal boxes twice weekly with food for 2 meals/day. Meals tailored according to recommended food items to increase, decrease or exclude (per nutrition information collected at baseline) with different functional ingredients added according to participants’ dietary metabolome. Participants also enrolled to a behavioural program via app and received prompts according to individual behaviours assessed by questionnaire at baseline, and inputs from the research team. | The control group received meal boxes twice weekly with food for 2 meals/day. Meals were visually identical to those provided to the intervention group but were not individualised. Control participants also received generic behavioural messaging via app. | No significant difference between groups in SBP (P=0.73), DBP (P=0.60). No significant difference in TC (P=0.81), HDL-C (P=0.35), LDL-C (P=0.63), TG (P=0.99) | No significant difference between groups in body weight (P=0.77), BMI (P=0.76), WC (P=0.67), fat mass (P=0.77), lean body mass (P=0.39), visceral adipose tissue (P=0.86), body total fat (P=0.74)) | Significantly improved intake of fibre in the intervention group (+7.8g/day (95% CI [2.4 to 13.2], P<0.01). No significant difference between groups in intake of energy (P=0.15), fat (P=0.85), SFA P=0.60), PUFA (P=0.40), MUFA (P=0.67), protein (P=0.21), carbohydrate (P=0.42), added sugar (P=0.74). | The personalised dietary plan informed by a combination of omics technologies did not yield greater benefits on blood pressure, blood lipids, body composition or diet beyond those obtained by a one-diet-fits-all approach. |
| Ammerman et al., 2003, USA | High cholesterol | Baseline n=468, 3 month follow-up n=407, 6 month follow-up n=343, 12 month follow-up n=349 (mean age 55.00, 71% F) | Parallel, 12 months | Blood lipids (TC, TG, HDL-C, LDL-C), anthropometry (weight), diet quality (DRA score) | Intervention: nurse with possible nutritionist referral, Control: nurse | Control group nurses were instructed to provide counselling for high cholesterol as they usually do. In addition, the Dietary Risk Assessment (DRA) instrument, without accompanying educational and counselling materials, was made available to control group nurses to use at their discretion. | A public health nurse directed component using the Food For Heart Program (tailored for low-income patients with high blood cholesterol, guided by dietary assessment of patients) during 3 counselling visits, with referral to a nutritionist if lipids remained elevated at 3-month follow-up (for 3 visits to review progress and work to set new goals with patients), and a reinforcement program consisting of a phone call from the nurse and 2 newsletters for strategies to enhance dietary change. | No statistically significant between-group difference in TC at 3 months (P=0.9), 6 months, or 12 months (P=0.6), LDL-C at 3 months (P=0.5), 6 months or 12 months (P=0.5), TG at 3, 6 and 12 months (P>0.4), and HDL-C ad 3, 6 and 12 months (P>0.2) | Statistically significant between-group improvement in weight in the intervention group at 3 months (-0.86 kg (95% CI 0.3 to 3.4 [0.14 to 1.55], (P=0.022)) and 6 months (-0.95 kg (95% CI 0.1 to 4.1 [0.05 to 1.86], (P=0.04)). No statistically significant improvement maintained at 12 months (P=0.13) | Statistically significant improvement in DRA score at 3 months (-3.7 (95% CI [1.9 to 5.5], P=0.0006), and at 12 months (-2.1 [95% CI 0.8 to 3.5], P=0.005) in the intervention group compared with the control. | While there was no difference between groups for the primary endpoint of total cholesterol reduction at 3-month follow-up, intervention group participants reported greater improvement in dietary intake and experienced modest, but statistically significant greater weight loss. These findings suggest public health nurses in rural areas should offer dietary counselling to those identified with high blood cholesterol. |
| Chang et al., 2022, USA | Elevated SBP and BMI >25kg/m^2^ | Baseline n=187, follow-up n=156  *Intervention*: mean age 54.80 ± 13.2 yrs, 53% F. *Control*: mean age control 54.50 ± 13.0 yrs, 51% F. | Parallel, 12 weeks | Blood pressure (24-h SBP, awake SBP, sleep SBP, 24h DBP, awake DBP, sleep DBP, AOBP SBP, AOBP DBP), anthropometry (weight, waist circumference), diet quality (HEI score), diet intake (total energy, sodium, sodium density, potassium, potassium density, sodium/potassium molar ratio) | Intervention: dietitian, Control: not reported | Treatment as per control group, as well weekly phone calls from a dietitian (ranging 30-60 minutes initial and 15-20 minutes for each of the weekly follow-ups). Dietitians examined meal logs of participants and explored their goals and values as part of their support. | Lifestyle guidance per American Heart Association guidelines and access to web-based applications containing educational material and a platform for meal-logging. Personalised dietary suggestions given based on results from FFQ at baseline. | Significant between-group improvement in the intervention group in sleep SBP (−5.47mmHg [95%CI −9.16 to −1.79], P=0.004) and sleep DBP (−4.04mmHg [95% CI −6.35 to −1.73], P=0.001). No statistically significant between-group improvements in 24-h SBP (P=0.2), awake SBP (P=0.5), 24-h DBP (P=0.1), awake DBP (P=0.3) AOBP SBP (P=0.4) or AOBP DBP (P=1.0) | No significant differences in weight (P=0.1) or waist circumference (P=0.5) | No significant between-group differences in HEI total score (P=0.09), however the intervention group had significantly greater improvements in HEI subcategories, including total  vegetables (+0.34 [95%CI 0.03 to 0.65], P=0.03), greens and beans (+0.54 [95%CI 0.09 to 0.99], P=0.02), . A significant difference in the potassium density was also showed in the intervention group (+0.21 [95%CI 0.07 to 0.35], P=0.003). | Dietitian-support and minimal-support approaches to dietary counselling to lower blood pressure using web-based applications resulted in similar improvements, with greater improvements seen in the dietitian-support group for sleep SBP and DBP. |
| Delahanty et al., 2001, USA | Hypercholesterolaemia | Baseline n=90, 3 month follow-up n=88.  *Intervention*: mean age 49.00± 10.00 yrs, 33% F. *Control*: mean age 49.90 ± 9.00, yrs, 33% F. | Parallel, 6 months | Blood lipids (TC, LDL-C, HDL-C, TG), anthropometry (weight), dietary intake (energy, fat %E, SFA, MUFA, PUFA, cholesterol, dietary fibre) | Intervention: dietitian, Control: physician | Received cholesterol-lowering nutritional counselling and treatment according to a NCEP-based protocol. Participants were required to meet with dietitians >2-3 times. If lipids were not in target range after initial treatment, participants had additional 2-3 follow-ups to provide a 6-month diet intervention. The number of visits based on an assessment of each participant’s eating habits, lifestyle, capabilities and motivation for change. Participants also maintained usual care from physicians. | Participants received customary cholesterol-lowering advice from healthcare provider which did not include contact with a dietitian. Participants agreed not to use lipid-lowering drugs or seek dietary counselling or therapy during intervention period. After intervention period, participants could commence hypolipidemic medication at their physician’s discretion. | Significant between-group improvements in intervention group in decrease in TC at 3 months (P<0.001) and 6 months (P<0.05), decrease in LDL-C at 3 months (P<0.05), decrease in HDL-C at 3 months (P<0.01) and decrease in TG at 3 months (P<0.05). No significant between group improvements in TC at 12 months (P>0.05), LDL-C at 6 or 12 months (P>0.05), HDL-C at 6 and 12 months (P>0.05), or TG at 6 or 12 months (P>0.05).  *NB: Magnitude of change between-groups not reported* | Significant between-group improvements in the intervention group in weight loss at 3 months (P<0.001) and 6 months (P<0.001). No significant between-group changes at 12 months (P>0.05).  *NB: Magnitude of change between-groups not reported* | Statistically significant between-group improvement in the intervention group in total energy at 3-months (P<0.05), % fat at 3 months (P<0.001) and 6 months (P<0.01), % SFA at 3 months (P<0.001) and 6 months (P<0.001), decrease in %MUFA at 3 months (P<0.01) and 6 months (P<0.01), decrease in cholesterol (mg/d) at 6 months (P<0.05) and increase in dietary fibre (g/d) at 3 months (P<0.05)  *NB: Magnitude of change between-groups not reported* | MNT was more effective at reducing blood lipids than usual care by a physician. MNT also led to greater weight loss and reduction of dietary fat intake than usual care. |
| Green et al., 2014, USA | Elevated BP, and Framingham CVD risk score between 10% and 25% and BMI >26kg/m2 | Baseline n=101, follow-up 90.  *Intervention*: mean age 55.90 ± 7.20 yrs, 53% F. *Control*: 57.80 ± 6.7 yrs, 30% F. | Parallel, 6 months | Blood pressure (SBP, DBP), blood lipids (TC, LDL-C, HDL-C) anthropometry (weight), CVD risk score (FRS), dietary intake (fruit, vegetables) | Intervention: dietitian, Control: not reported | Provided with same information as control as well as a scale, pedometer and home BP monitor for self-monitoring. Dietitian reviewed patient’s lifestyle habits, usual diet and baseline laboratory measurements and generated an estimated “heart age.” Participants educated on DASH diet and goals to improve blood pressure, lipid targets and weight loss or maintenance during initial in-person visit. Next dietitian helped to create personalised action plans, then maintained contact via EHR-linked secure messaging, where self-monitoring data were shared. Participants were asked to share their self-monitoring data with the dietician at least once a week for 2 months, followed by every 2 weeks for 2 months, and then every month for 2 months, for a total of 6 months. Dietitians used secure messaging to respond to questions and provide resources and to encourage participants to reach their goals. | Participants informed at baseline that their BP was high, encouraged to follow-up with physician for usual care. They received copies of their laboratory results via patient website and by mail. | No statistically significant between-group differences in SBP (P=0.40), DBP (P=0.32) TC (P=0.52), LDL-C (P=0.71), HDL-C (P=0.24) | Statistically significant between-group improvement in weight in intervention group (−3.2kg [95% CI −5.0, −1.5], P<0.01) | Statistically significant between-group improvement in fruits and vegetables in intervention group (+2.3 serves/day [95%CI 1.2, 3.3], P<0.01)) | The trial demonstrated similar results in usual care and dietitian-support arms, with greater improvements in weight loss and dietary intake observed in the intervention group. The results demonstrated promising BP/CVD risk-reduction outcomes. |
| Henkin et al., 2000, Israel | Hyperlipidaemia | Baseline n=136, follow-up n=118 *Intervention*: mean age 51.00 ± 11.00 yrs, 11% F. *Contro*l mean age: 50.00±11.00 yrs, 6% F | Parallel, 3 months | Blood lipids (TC, TG, LDL-C, HDL-C, TC/HDL-C). Coronary heart disease risk category, BMI and dietary intake (baseline only) | Intervention: physician and dietitian, Control: physician | Participants received 2 to 4 individual counselling sessions, dictated by their own progress. Counselling by the dietitians differed from that by the physicians by including a more detailed analysis of eating habits and participants were provided more specific dietary recommendations. As necessary, progression to Step II diet was offered to participants. | Counselling by physicians performed in-person for 30 minutes, where patient risk factors were evaluated, and counselling was performed about smoking cessation, physical activity, weight control, and dietary modifications for the Step I diet. Educational literature, including general information about hypercholesterolemia, guidelines for healthy nutrition, as well as some specific dietary recommendations, was given to the participants. The 3-day food diaries were available to the physician during this evaluation if specifically requested. | Significant between-group improvements in intervention group in TC at 6 weeks (-4mg/dL [95% CI 0 to 7], P<0.05), 3 months (-4mg/dL [95%CI 1 to 7], P<0.05), and 1 year (-4mg/dL [95% CI 0 to 8], P<0.05), in LDL-C at 3 months (-5mg/dL [95%CI 1 to 9], (P<0.05) and in TC/HDL-C ratio at 1 year (-8 [95%CI 0 to 13], P<0.05). Nil significant between-group differences in TC at 6 months (P>0.05), LDL-C at 6 weeks, 6 months or 12 months (P>0.05), HDL-C at 6 weeks, 3 months, 6 months or 12 months (P>0.05) or TG at 6 weeks, 3 months, 6 months or 12 months (P>0.05 | Not reported | Not reported | Dietary counselling by dietitians led to more significant improvements in blood lipids at 3 months than the physician group, however, during long-term follow up, these results gradually increased in both groups. In future studies, a modified dietary approach, possibly combined with earlier introduction of lipid-lowering medications, should be considered in hypercholesterolemic patients. |
| Hjerkinn et al., 2005, Norway | High cholesterol and high SBP | Baseline n=563, 3-year follow-up n=487  (age range 64-76 yrs, mean age 70. 100% M) | Parallel, 3 years | Blood pressure (SBP, DBP [baseline only])  Blood lipids (TC, HDL-C. LDL-C, TC/HDL-C, TG), anthropometry (BMI and waist-hip ratio [baseline only]), dietary intake (total energy, carbohydrate %E, protein %E, fat %E, SFA, MUFA, PUFA, n-3 PUFA, n-6 PUFA, fibre) | Intervention: nutritionist, Control: not reported | In two study arms, dietary counselling given face-to-face for first visit (30-45 minutes) and visit at 3 months. After 3 months, telephone follow-ups every 6 months (30 minutes). Advice individualised on the basis of a food frequency questionnaire at baseline. Special oil and margarine provided to participants to decrease saturated fat intake from animal sources. In one of these study arms, n-3 supplementation was also provided. | No dietary intervention in the two control groups, however, one group was supplemented with n-3 fatty acids. | No significant between-group improvement in the dietary intervention arms compared to the control arms for TC (P>0.05), HDL-C (P>0.05), or TC:HDL ratio (P>0.05). Significant between group improvements in dietary groups for TGs (P=0.003).  *NB: Magnitude of change between-groups not reported* | Significant between-group improvement in the dietary intervention arms compared to the control arms for decrease in BMI (P=0.005)  *NB: Magnitude of change between-groups not reported* | Significant between-group improvement in intervention arms compared to control group arms in increased intake of carbohydrates %E (P=0.001), decreased intake of fat %E (P<0.001), decreased intake of SFA (P<0.001), decreased intake of MUFA (P=0.007), increased intake of PUFA (P=0.045), decreased intake of n-3 fatty acids (P=0.012), and increased intake of fibre (P<0.001). No significant between-group improvement in total energy intake (P>0.05), protein intake (P>0.05), or n-6 fatty acids (P>0.05)  *NB: Magnitude of change between-groups not reported* | Dietary counselling did not lead to any changes in TC, and showed non-significant changes in HDL-C. Dietary counselling was associated with weight loss and improved dietary quality, however, n-3 supplementation intervention arms saw similar improvements in weight loss. The authors conclude that in the DOIT study population, it was feasible and possible to modify dietary habits and serum fatty acid profile by intervention with dietary counselling, n-3 PUFA supplementation, or both. |
| Kucharska et al., 2018, Poland | Overweight/ obese with hypertension | Baseline n=126, follow-up n=121  *Intervention*: mean age 61.34 ± 7.90 yrs, 30% F. *Control*: mean age 58.11 ± 8.52 yrs, 30% F. | Parallel, 3 months | Blood pressure (SBP, DBP), anthropometry (weight, BMI, waist circumference, body fat% and body fat content [kg]) | Intervention: researcher Control: not reported | Provided with an individualised, three-month diet plan, three subsequent individual counselling appointments as well as a lecture in groups of four to six. Principles of the DASH diet, risk factors for hypertension, and a focus on excessive weight reduction were discussed. | Standard advice | Significant between-group improvements in the intervention group in SBP (-3.79mmHg, P=0.000) and DBP -4.38mmHg, P=0.002) | Significant between-group improvements in the intervention group in body weight (-4.74kg, P=0.000) and BMI (-1.8kg/m^2^, P=0.005), waist circumference (-5.71cm, P=0.000), body fat % (-1.97%, p<0.001) and body fat content (-3.81kg, P<0.0001) | Not reported | An individualised dietary intervention based on DASH diet principles was found to be effective at improving blood pressure and body composition outcomes in overweight/obese adults with primary hypertension. Future research is needed to evaluate long-term efficacy. |
| Lee et al., 2021, Korea | NAFLD taking antihyperlipidemic medications. | Baseline n=64, follow-up n=48  *Intervention*: 42.00 ± 12.00 yrs, 33.4% F.  *Control*: 39.00 ± 11.00 yrs, 43.0% F | Parallel, 48 weeks | Blood lipids (TC, LDL-C, HDL-C, TG) and anthropometry (BMI, %body fat, muscle mass) | *Intervention*: Nutritionist, *Control*: not reported | The MNT group were provided with nutrition education specific for NAFLD and personalised based on a 24-hour recall at baseline. Face-to-face education conducted 5 times, at 0, 12, 24, 36 and 48 weeks. Monthly phone monitoring was also conducted to evaluate the level of dietary practice and give feedback to patients. | Control participants provided with a brochure on dietary information for NAFLD patients. | No significant between-group difference observed in TC (P=0.392), LDL-C (P=0.456), HDL-C (P=0.767) or TG (P=0.381) | No significant between-group difference observed in BMI (P=0.974), muscle mass (P=0.427), %body fat (P=0.261). | Not reported | There was no evidence from the present study to support the added benefit of MNT to antihyperlipidemic pharmacotherapy for blood lipids or BMI. |
| Lim et al., 2008, Korea | Hyperlipidaemia | Baseline and follow-up n=40 *Intervention*: 46.70 ± 6.50 yrs, 45% F.  *Control*: 43.40 ± 10.70 yrs, 40% F. | Parallel, 12 weeks | Blood lipids (TC, LDL-C, LDL-C, HDL-C), blood pressure (SBP, DBP), anthropometry (weight, BMI, WC, W/H ratio) and dietary intake (total energy, C/P/F ratio, fibre, cholesterol, folate, vitamin B6) | *Intervention*: Dietitian, *Control*: not reported | A registered dietitian performed MNT for participants 5 times (ranging from 30–40-minute appointments) during the study, based on guidelines for Korean hyperlipidaemic patients. MNT was based on evaluation of nutritional status, anthropometric and blood analysis from baseline. Follow-up feedback was also provided by the dietitian, aimed at reinforcing the messages of the intervention. | No intervention | Between-group differences not reported. | Between-group differences not reported. | Between-group differences not reported. | This study found intensive MNT effective in improving the blood lipid profiles of Korean hyperlipidaemic patients. The MNT group also saw improvements in BMI, as well as some improvements in dietary intake. |
| Miller et al., 2016, USA | Controlled hypertension | Baseline and follow-up n=123  *Intervention*: 58.80 ± 8.70 yrs, 66% F.  *Control*: 58.50 ± 10.4yrs, 75% F. | Parallel, 8 weeks | Blood pressure (SBP, DBP), blood lipids (TC, TG, LDL-C, HDL-C), anthropometry (weight), dietary intake (servings of fruits and vegetables, potassium, magnesium, vitamin C, fibre, fat %E, cholesterol) | *Intervention*: Study coach (using Dietitian-developed material), *Control*: Study coordinator | Participants received single 1-hour in-person session with study coach on adoption of the DASH diet, followed by weekly 15-minute phone calls. The intervention also included a $30/week allowance for purchasing high-potassium foods, chosen from a pre-determined list. The coach provided education and recommendations specific to the participant’s needs, preferences and local context, and used literacy-sensitive modules about hypertension to guide the discussions. | Control participants received printed material on improving BP control using the DASH diet in a one-off, 15-minute visit with the study coordinator. Participants received a total of $240 to purchase foods at the same supermarket as the intervention group, but no advice was given. | No significant between-group differences in SBP (P=0.48), DBP (P=0.33), TC (P=0.80), TG (P=0.29), LDL-C (P=0.82), HDL-C (P=0.84) | No significant between-group differences in weight (P=0.51) | Significant difference in change between-groups was observed, with the intervention group showing more favourable results for the following variables: intake of fruits and vegetables (+1.4 serves/day [95%CI 0.7, 2.1], P<0.001), potassium (+0.42g/day [95%CI 0.12, 0.71], P=0.007), magnesium (+42mg/day [95%CI 12, 72], P=0.007), vitamin C (+24mg/day [95%CI 7, 41], P=0.007), and fibre (2.8g/day [95%CI 0.8, 4.8], P=0.007). No significant between-group differences observed in fat %E (P=0.25) or dietary cholesterol (P=0.25) | The results were not able to demonstrate a significant effect on BP in African American patients, possibly due to an insufficient number of BP measurements and changes to medications post-randomisation. The intervention, however, led to significant improvements in dietary intake. |
| Noda et al., 2012, Japan | Hypertension and/or diabetes mellitus | Baseline n=200, 4-week follow-up n= 191, 8 week-follow up n=187.  *NB*: There were four groups with different combinations of dietary counselling and delivered meals: group A (no counselling during either study period), group B (counselling with the ordinary diet, but not with delivered meals), group C (counselling with delivered meals, but not with the ordinary diet), and group D (counselling for both study periods).  *Intervention*: groups (received some kind of dietary counselling)  [B]: 64.00±12.50yrs, 58% F,  [C]: 65.90±12.70yrs, 42%,  [D]: 64.50±10.60yrs,  62% F.  *Control group*  [A]: 65.60±11.40yrs, 44% F. | Parallel, 8 weeks | Blood pressure (SBP, DBP), blood lipids (TG, HDL-C, LDL-C), anthropometry (weight, WC) | *Intervention*: Dietitian *Control*: Physician | Participants received individual face-to-face dietary counselling for 30-60 minutes based on information on existing dietary habits collected at baseline. The counselling focused on good nutrition, meal planning, dietary calories, and alcohol consumption. Another 10-20-minute phone call was made during the intervention period to review dietary performance and advise participants, with a maximum of 2 counselling sessions for groups B and C and 4 for group D. | Control group participants received no intervention for the first 4 weeks of the study, then calorie-controlled meals were provided for the second half, with no further dietary intervention. | Significant group*period interaction effect for SBP and DBP was noted. Those who received dietary counselling at some point throughout the study (combined groups B+C+D) had significant reductions in SBP (Estimated SE -2.02±0.74mmHg, P= 0.007) and DBP (Estimated SE -1.19±0.41mmHg, P= 0.004), whereas group A participants did not (Estimated SE 2.03±1.31mmHg, P= 0.12 for SBP and Estimated SE 0.32±0.72mmHg, P =0.66 DBP).  Not significant difference noted for TG across groups at various time points (0, 4 and 8 weeks). | Significant group*period interaction effect was found for WC, where combined groups B+C+D (Estimated SE –0.33±0.07kg, P <0.001) saw significant reductions, but group A did not (Estimated SE –0.01±0.11 kg, P=0.34).  Significant between-group differences, with a greater reduction in body weight in those who received dietary counselling (combined groups B+C+D) versus those who did not receive dietary counselling (group A), P = 0.03. | Not reported. | Counselling by dietitians and delivery of calorie-controlled meals were effective for reducing body weight and blood pressure in patients with HT and/or T2DM. |
| Piernas et al., 2020, UK | High cholesterol | Baseline n=113, follow-up n=106  *NB:* This study included three groups, a control group [C] that received no intervention, the brief support [BS] group, which received generic advice, and the brief support plus personalised food shopping feedback [BS+SF]  *Intervention*: BF+SF: 59.9±11.7yrs, 66.7% F  *Control groups:* BS: 64.7±9.2yrs, 70.8 % F,  C: 62.9±11.2yrs,  64.7% F | Parallel, 3 months | Blood lipids (TC, TG, LDL-C, HDL-C, non-HDL-C, TC/HDL ratio), blood pressure (SBP, DBP) anthropometry (weight), dietary intake (total energy, fat %E, SFA %E, PUFA %E, MUFA %E, fibre) | *Intervention*: [BS+SF] nurse/healthcare practitioner *Control* (brief support [BS] group) nurse/healthcare practitioner, *Control* [C] (no intervention) not reported | Intervention group participants received brief support per the BS group. In addition, they received shopping reports based on SFA in their weekly shop at baseline and the end of the first, second and third month of the intervention. The reports showed mean weekly SFA content of shopping and identified main contributors to SFA over the previous period, as well as suggestions for swaps to foods containing less SFA. | Control group participants received no intervention, were informed of the results of their blood tests and invited to a further check after 3 months. The BS group received a single 10-minute appointment with a nurse or healthcare practitioner, where participants were informed of the effects of SFA on cholesterol and provided tips to reduce SFA guided by the NHS website. | Significant between-group difference in BS+SF group compared to C group in HDL-C (−0.13mmol/L [95% CI −0.26, 0.00], P=0.047). No significant between-group differences in BS+SF group compared to C in LDL-C (P=0.790), TC (P=0.910), TG (P=0.211), non-HDL-C (P=0.393), TC/HDL ratio (P=0.335), SBP (P=0.710) or DBP (P=0.997) Significant between-group change in BS+SF group compared to BS in TC (+0.28mmol/L [95% CI 0.04, 0.51], P=0.02), non-HDL-C (+0.31mmol/L [95% CI 0.06,  0.56], P= 0.016), and TC/HDL ratio (+0.38 [95% CI 0.01-0.75], P=0.042). Nil significant between group improvements in BS+SF group compared to BS in LDL-C (P=0.095), HDL-C (P=0.713), TG (P=0.154), SBP (P=0.759) or DBP (P=0.397) | Nil significant between-group differences in weight in BS+SF group compared to control (P=0.43) or BS (P=0.449) | Significant between-group improvement in PUFA intake in BS+SF compared to BS (1.2%EI [95%CI 0.2,2.1], P=0.016), but not compared to C group (P=0.076). No significant between-group differences in BS+ SF group in energy, total fat, SFA, MUFA or fibre intakes compared to C or BS groups. | Among people identified as having modestly raised LDL-C, there was no strong evidence that a brief behaviourally informed appointment with a HCP with or without additional personalised shopping feedback and swap suggestions for the saturated fat content of food purchases reduced SFA consumption, SFA food purchases, or LDL-C relative to a control group. While the reductions in SFA and LDL cholesterol in the intervention groups were not significant they were larger than those in the control group with no formal intervention. |
| Willaing et al., 2004, Denmark | High risk IHD: hyperlipidaemia/elevated BMI/familial CVD risk/high waist circumference/high blood pressure/T2DM | Baseline n=503, follow-up n=339 *Intervention*: 50yrs (range 18-87yrs), 71% F.  *Control:*  54yrs (range 18-84yrs), 66% F. | Parallel, 12 months | Blood lipids (TC, LDL-C, HDL-C, TG) CVD risk score (CRS), blood pressure (SBP, DBP [baseline only]), anthropometry (weight, BMI, waist circumference) | *Intervention*: Dietitian, *Control*: Physician | Five individual counselling sessions with dietitian based on indication for referral, diet history and diet routines. Counselling focused on principles of good nutrition, food shopping, meal planning and exercise. Sessions lasted 1 hour initially with 30-minute follow-ups | Five counselling sessions with physician on IHD risk factors. Initial session 30 minutes, followed by 12-minute appointments. Counselling consisted of general advice and delivery of commercially available information on healthy diet. | Statistically significant between-group difference, with an increase in control group noted for HDL-C versus intervention group (+0.10mmol/L, P=0.003) and a reduction in CRS (-0.1194, P=0.01) No significant between-group differences in TC (P=0.57), LDL-C (P=0.72), TG (P=0.11) | Statistically significant between-group improvements in intervention group compared to control in weight (-2.1kg, P=0.02) and BMI (-0.72kg/m^2^, P=0.02). No statistically significant difference observed in waist circumference (P=0.24) | Not reported | A GP or a dietitian could provide nutritional counselling in general practice with slightly different outcomes regarding weight reduction and change in lipids. GPs more effectively obtained risk reduction in relation to risk of IHD. |
| Wong et al., 2015, China | Hypertension (grade 1) | Baseline n=556, 6-month follow-up n=504, 12-month follow-up n=485  *Intervention:*  55.40±5.60yrs, 46.4% F  *Control:*  54.90±5.20yrs, 48.4% F. | Parallel, 12 months | Blood lipids (TC, LDL-C, HDL-C, TG), blood pressure (SBP, DBP), anthropometry (BMI), dietary intake (intake of food groups including vegetables, fruit, grains, dairy, meat, poultry, fish and eggs, nuts, seeds and legumes, fats and oils, and sweets). | *Intervention*: Dietitian  *Control*: Physician | Dietary counselling on DASH diet in a once-off 25 minute in-person counselling session plus general advice from physician. Individualised meal plans and counselling based on Chinese culture and individual diet goals. | Usual care advice by physician provided in-person based on standard pamphlet for HT patients. | No significant difference between groups in TC at 6 months (P=0.55 or 12 months (P=0.91), LDL-C at 6 months (P=0.70) or 12 months (P=0.88), HDL-C at 6 months (P=0.10) or 12 months (P=0.34) or TG at 6 months (P=0.60) or 12 months (P=0.84).  No significant difference between groups in SBP at 6 months (P=0.54) or 12 months (P=0.94) or DBP at 6 months (P=0.24) or 12 months (P=0.20). | No significant difference between groups for BMI at 6 months (P=0.08) or 12 months (P=0.49). | Significantly improved intake of vegetables in the intervention group at 12 months (+0.46 serves/day [95%CI 0.04 to 0.88], P=0.032) and dairy at 12 months (+0.09 serves/day [95%CI 0.01 to 0.16], P=0.021) and meat, poultry, fish and eggs at 6 months (-0.70 serves/day [95%CI -1.32 to -0.09], P=0.026).  No significant difference between groups for intake of grains at 6 months (P=0.456) or 12 months (P=0.674), vegetables at 6 months (P=0.442), fruits at 6 months (P=0.352) or 12 months (P=0.643), dairy at 6 months (P=0.077), meat, poultry, fish and eggs at 12 months (P=0.592), nuts seeds and legumes at 6 months (P=0.702) or 12 months (P=0.648), fats and oils at 6 months (P=0.513) or 12 months (P=0.488) or sweets at 6 months (P=0.354) or 12 months (P=0.148). | Decreases in clinical outcomes and anthropometric measures with a similar magnitude were observed between both the intervention and control groups, therefore physicians may still practice usual care to reduce unnecessary referrals to dietitians for counselling. Further explorations on different DASH delivery models are warranted to inform clinical practice. |
| Wong et al., 2016, China | Hypertension (grade 1) | Baseline n=556, 6-month follow-up n=504, 12 month follow-up n=485  *Intervention:*  55.40±5.00yrs  53.4% F  *Control:*  54.70±5.00yrs, 48.4% F. | Parallel, 12 months | 10-year CVD risk score | *Intervention*: Dietitian *Control*: Physician | Dietary counselling on DASH diet in a once-off 25 minute in-person counselling session plus general advice from physician. Individualised meal plans and counselling based on Chinese culture and individual diet goals. | Usual care advice by physician provided in-person based on standard pamphlet for HT patients. | No significant difference between groups in CVD risk score at 6 months (P=0.477) or 12 months (P=0.568) | Not reported | Not reported | The automatic referral of newly diagnosed grade 1 hypertensive patients for one-to-one dietitian counselling in a single session may not be supported based on this evidence, as similar improvements were seen in both intervention and control groups. |

AOBP; Automated office blood pressure; BMI, Body mass index; BP, Blood pressure; CRS, Copenhagen Risk Score; CVD, Cardiovascular disease; DASH, Dietary approaches to stop hypertension; DBP, Diastolic blood pressure; DRA, Dietary risk assessment; EHR, Electronic health record; FRS, Framingham Risk Score; HCP, Health care practitioner, HDL-C, High-density lipoprotein cholesterol; HEI, Healthy Eating Index; HT, hypertension; IHD, Ischaemic heart disease; LDL-C, Low-density lipoprotein cholesterol; MUFA, Monounsaturated fatty acids; NAFLD, Non-alcoholic fatty liver disease; NCEP, National Cholesterol Education Program; n-3, Omega-3; n-6, Omega-6; PUFA, Polyunsaturated fatty acids; SBP, Systolic blood pressure; SFA, Saturated fatty acids; T2DM, Type 2 diabetes mellitus; TC, Total cholesterol; TG, Triglycerides; %E, %energy; WC, waist circumference.

**Supplementary Table 4** Academy of Nutrition and Dietetics Quality Assessment Criteria Checklist

| Study reference | Relevance questions  **Q1         Q2         Q3         Q4** | | | | Validity questions  **Q1          Q2         Q3         Q4          Q5          Q6         Q7          Q8          Q9        Q10** | | | | | | | | | | Overall rating |
| --- | --- | --- | --- | --- | --- | --- | --- | --- | --- | --- | --- | --- | --- | --- | --- |
| Wong et al, 2015 | Yes | Yes | Yes | Yes | Yes | Yes | Yes | Yes | Yes | Yes | Yes | Yes | Yes | Yes | (+) |
| Wong et al, 2015 | Yes | Yes | Yes | Yes | Yes | Yes | Yes | Yes | Yes | Yes | Yes | Yes | Yes | Yes | (+) |
| Aldubayan et al, 2022 | Yes | Yes | Yes | Yes | Yes | Yes | Yes | Yes | Yes | Yes | Yes | Yes | Yes | Yes | (+) |
| Kucharska et al, 2018 | Yes | Yes | Yes | Yes | Yes | Yes | Yes | Yes | No | No | Yes | Yes | Yes | ? | (+) |
| Chang et al, 2022 | Yes | Yes | Yes | Yes | Yes | Yes | Yes | No | Yes | Yes | Yes | Yes | Yes | Yes | (+) |
| Green et al, 2014 | Yes | Yes | Yes | Yes | Yes | Yes | Yes | No | No | Yes | Yes | Yes | Yes | ? | (+) |
| Ammerman et al, 2003 | Yes | Yes | Yes | Yes | Yes | Yes | Yes | Yes | Ye | Yes | Yes | Yes | Yes | ? | (+) |
| Delahanty et al, 2000 | Yes | Yes | Yes | Yes | Yes | Yes | Yes | Yes | No | No | Yes | Yes | No | No | (+) |
| Hjerkinn et al, 2005 | Yes | Yes | Yes | Yes | Yes | Yes | Yes | Yes | Yes | Yes | Yes | Yes | No | No | (+) |
| Miller et al, 2016 | Yes | Yes | Yes | Yes | Yes | Yes | Yes | Yes | Yes | Yes | Yes | Yes | Yes | Yes | (+) |
| Lee et al, 2010 | Yes | Yes | Yes | Yes | Yes | Yes | Yes | Yes | Yes | Yes | Yes | Yes | Yes | Yes | (+) |
| Lim et al, 2008 | Yes | Yes | Yes | Yes | Yes | Yes | Yes | No | No | Yes | Yes | Yes | Yes | ? | (+) |
| Noda et al, 2012 | Yes | Yes | Yes | Yes | Yes | Yes | Yes | Yes | Yes | Yes | Yes | Yes | Yes | ? | (+) |
| Piernas et al, 2020 | Yes | Yes | Yes | Yes | Yes | Yes | Yes | Yes | Yes | Yes | Yes | Yes | Yes | Yes | (+) |
| Henkin et al, 2000 | Yes | Yes | Yes | Yes | Yes | Yes | Yes | Yes | No | Yes | Yes | Yes | Yes | No | (+) |
| Willaing et al, 2004 | Yes | Yes | Yes | Yes | Yes | Yes | ? | No | No | Yes | Yes | Yes | Yes | Yes | (+) |

?: Unclear. +: Positive. ϴ: Neutral. -: Negative. n/a: not applicable.

Quality Criteria Checklist Questions

**Relevance**

**1. Would implementing the studied intervention or procedure (if found successful) result in improved outcomes for the patients/clients/population group? (NA for some epidemiological studies)**

**2. Did the authors study an outcome (dependent variable) or topic that the patients/clients/population group would care about?**

**3. Is the focus of the intervention or procedure (independent variable) or topic of study a common issue of concern to dietetics practice?**

**4. Is the intervention or procedure feasible? (NA for some epidemiological studies)**

**Validity**

**1. Was the research question clearly stated?** 
1.1 Was the specific intervention(s) or procedure (independent variable(s)) identified? 
1.2 Was the outcome(s) (dependent variable(s)) clearly indicated?

1.3 Were the target population and setting specified?

**2. Was the selection of study subjects/patients free from bias?**

2.1 Were inclusion/exclusion criteria specified (e.g., risk, point in disease progression, diagnostic or prognosis criteria), and with sufficient detail and without omitting criteria critical to the study?

2.2 Were criteria applied equally to all study groups?

2.3 Were health, demographics, and other characteristics of subjects described?

2.4 Were the subjects/patients a representative sample of the relevant population?

**3. Were study groups comparable?**

3.1 Was the method of assigning subjects/patients to groups described and unbiased? (Method of randomization identified if RCT)

3.2 Were distribution of disease status, prognostic factors, and other factors (e.g., demographics) similar across study groups at baseline?

3.3 Were concurrent controls used? (Concurrent preferred over historical controls.)

3.4 If cohort study or cross-sectional study, were groups comparable on important confounding factors and/or were preexisting differences accounted for by using appropriate adjustments in statistical analysis?

3.5 If case control study, were potential confounding factors comparable for cases and controls? (If case series or trial with subjects serving as own control, this criterion is not applicable. Criterion may not be applicable in some cross-sectional studies.)

3.6 If diagnostic test, was there an independent blind comparison with an appropriate reference standard (e.g., “gold standard”)?

**4. Was method of handling withdrawals described?**

4.1 Were follow up methods described and the same for all groups?

4.2 Was the number, characteristics of withdrawals (i.e., dropouts, lost to follow up, attrition rate) and/or response rate (cross-sectional studies) described for each group? (Follow up goal for a strong study is 80%.)

4.3 Were all enrolled subjects/patients (in the original sample) accounted for?

4.4 Were reasons for withdrawals similar across groups?

4.5 If diagnostic test, was decision to perform reference test not dependent on results of test under study?

**5. Was blinding used to prevent introduction of bias?**

5.1 In intervention study, were subjects, clinicians/practitioners, and investigators blinded to treatment group, as appropriate?

5.2 Were data collectors blinded for outcomes assessment? (If outcome is measured using an objective test, such as a lab value, this criterion is assumed to be met.)

5.3 In cohort study or cross-sectional study, were measurements of outcomes and risk factors blinded?

5.4 In case control study, was case definition explicit and case ascertainment not influenced by exposure status?

5.5 In diagnostic study, were test results blinded to patient history and other test results?

**6. Were intervention/therapeutic regimens/exposure factor or procedure and any comparison(s) described in detail? Were intervening factors described?**

6.1 In RCT or other intervention trial, were protocols described for all regimens studied?

6.2 In observational study, were interventions, study settings, and clinicians/provider described?

6.3 Was the intensity and duration of the intervention or exposure factor sufficient to produce a meaningful effect?

6.4 Was the amount of exposure and, if relevant, subject/patient compliance measured?

6.5 Were co-interventions (e.g., ancillary treatments, other therapies) described?

6.6 Were extra or unplanned treatments described?

6.7 Was the information for 6.4, 6.5, and 6.6 assessed the same way for all groups?

6.8 In diagnostic study, were details of test administration and replication sufficient?

**7. Were outcomes clearly defined and the measurements valid and reliable?**

7.1 Were primary and secondary endpoints described and relevant to the question?

7.2 Were nutrition measures appropriate to question and outcomes of concern?

7.3 Was the period of follow-up long enough for important outcome(s) to occur?

7.4 Were the observations and measurements based on standard, valid, and reliable data collection instruments/tests/procedures?

7.5 Was the measurement of effect at an appropriate level of precision?

7.6 Were other factors accounted for (measured) that could affect outcomes?

7.7 Were the measurements conducted consistently across groups?

**8. Was the statistical analysis appropriate for the study design and type of outcome indicators?**

8.1 Were statistical analyses adequately described the results reported appropriately?

8.2 Were correct statistical tests used and assumptions of test not violated?

8.3 Were statistics reported with levels of significance and/or confidence intervals?

8.4 Was “intent to treat” analysis of outcomes done (and as appropriate, was there an analysis of outcomes for those maximally exposed or a dose-response analysis)?

8.5 Were adequate adjustments made for effects of confounding factors that might have affected the outcomes (e.g., multivariate analyses)?

8.6 Was clinical significance as well as statistical significance reported?

8.7 If negative findings, was a power calculation reported to address type 2 error?

**9. Are conclusions supported by results with biases and limitations taken into consideration?**

9.1 Is there a discussion of findings?

9.2 Are biases and study limitations identified and discussed?

**10. Is bias due to study’s funding or sponsorship unlikely?**

10.1 Were sources of funding and investigators’ affiliations described?

10.2 Was there no apparent conflict of interest

**Supplementary Figure 1** Total Cholesterol (TC) Meta-analysis data


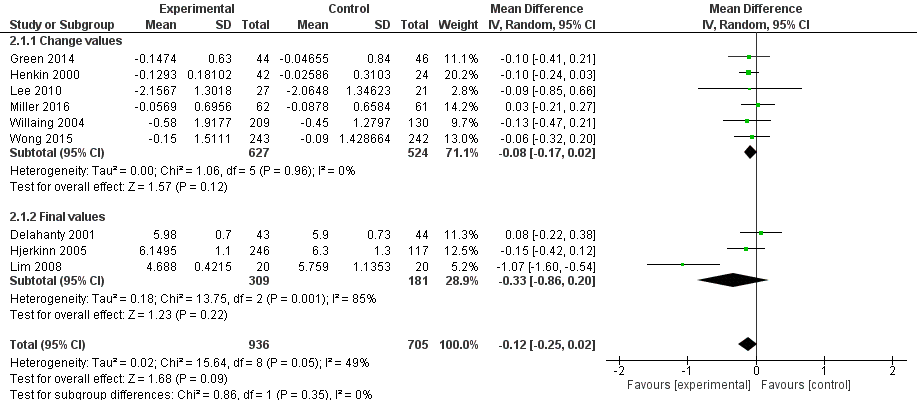


**Supplementary Figure 2** Low-Density Lipoprotein Cholesterol (LDL-C) Meta-analysis data

**
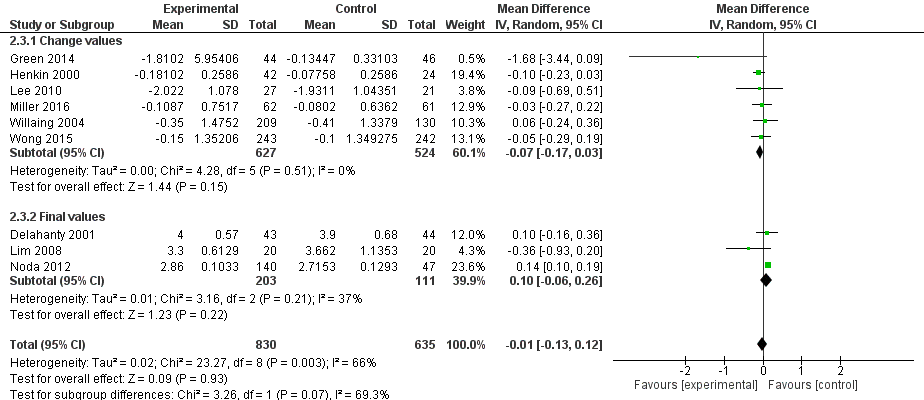
**

**Supplementary Figure 3** High-Density Lipoprotein Cholesterol (HDL-C) Meta-analysis data

**
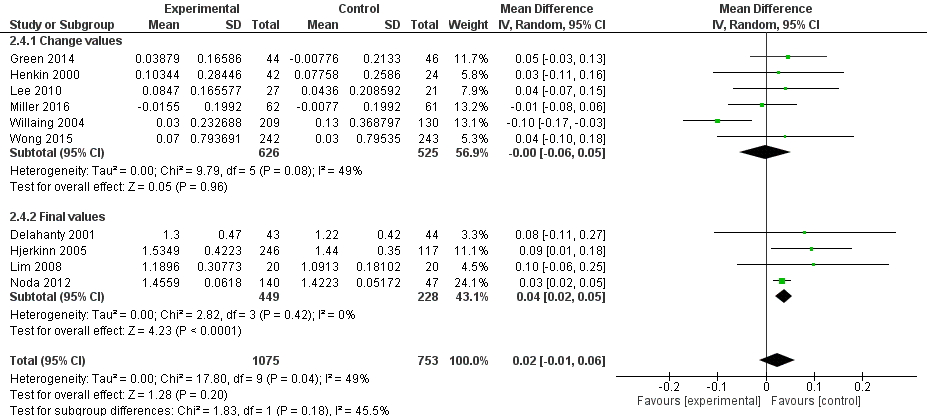
**

**Supplementary Figure 4** Triglycerides (TG) Meta-analysis data


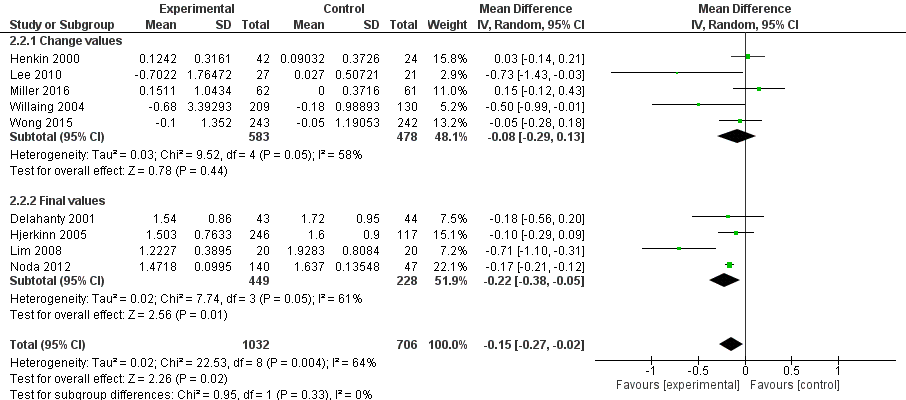


**Supplementary Figure 5** Weight Meta-analysis data


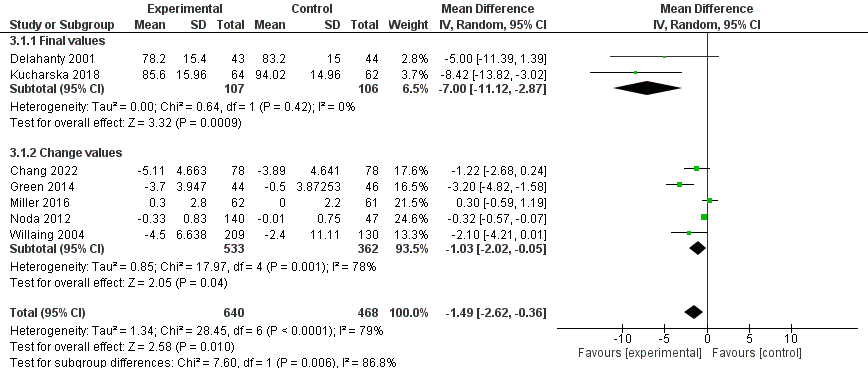


**Supplementary Figure 6** Body Mass Index (BMI) Meta-analysis data


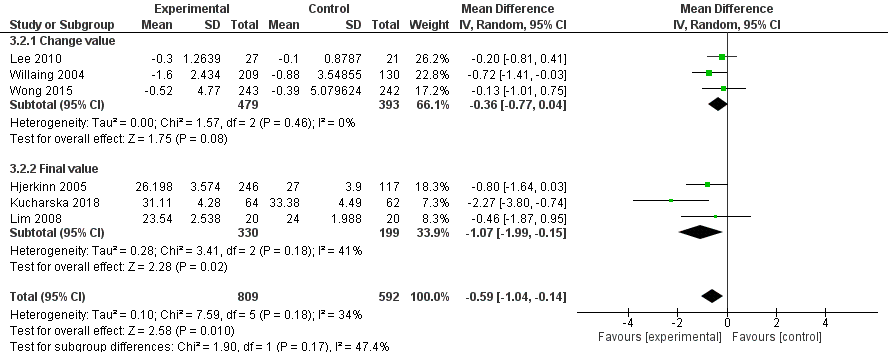


**Supplementary Figure 7**– Waist Circumference (WC) Meta-analysis data


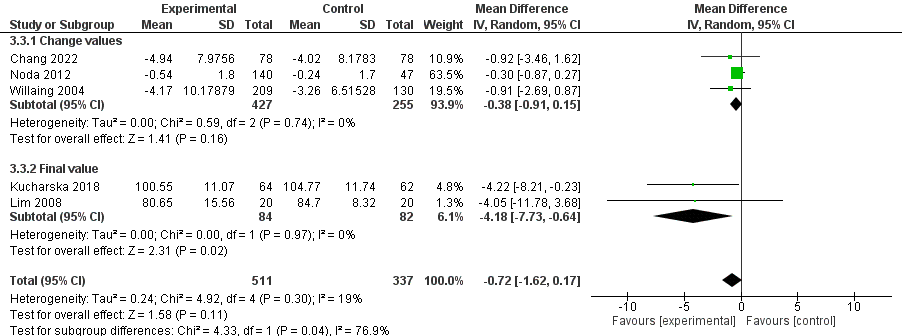


**Supplementary Figure 8** Total Cholesterol (TC) Dietitian sub-group analysis


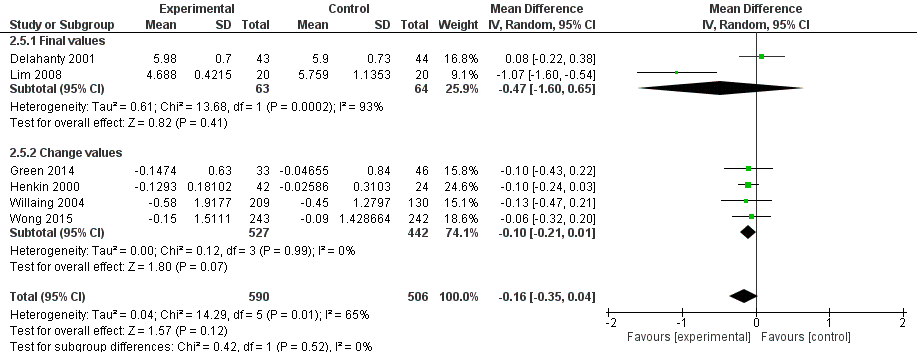


**Supplementary Figure 9** Low-Density Lipoprotein Cholesterol (LDL-C) Dietitian sub-group analysis


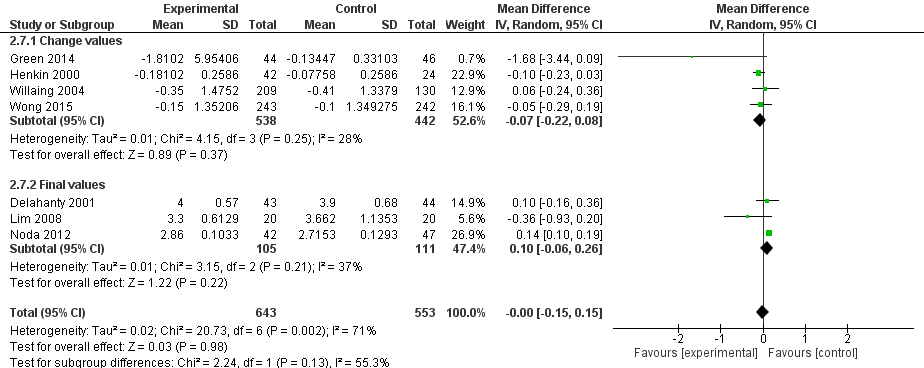


**Supplementary Figure 10** High-Density Lipoprotein Cholesterol (HDL-C) Dietitian sub-group analysis


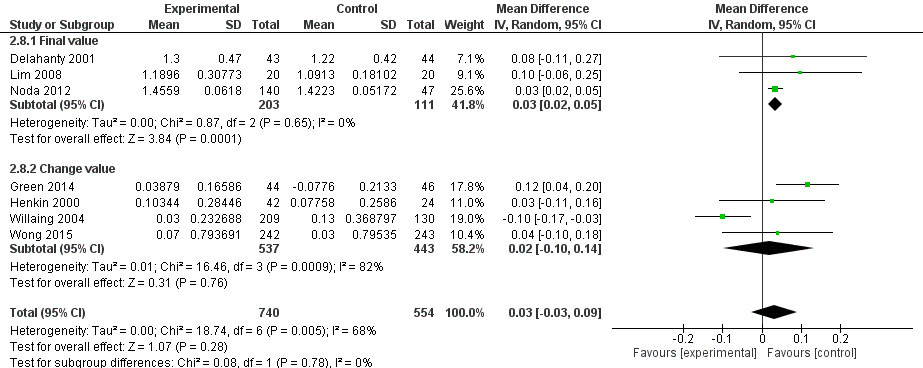


**Supplementary Figure 11** Weight - Dietitian sub-group analysis


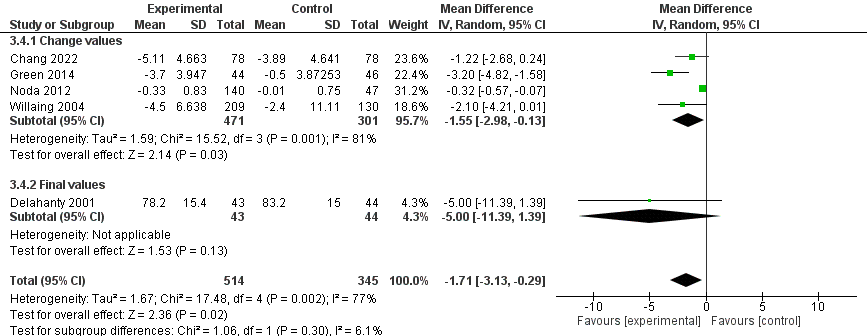


**Supplementary Figure 12** Body Mass Index (BMI) Dietitian sub-group analysis


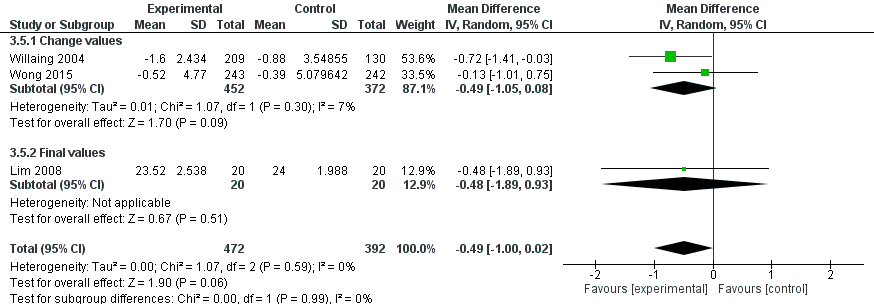


**Supplementary Figure 13** Waist Circumference (WC) Dietitian sub-group analysis


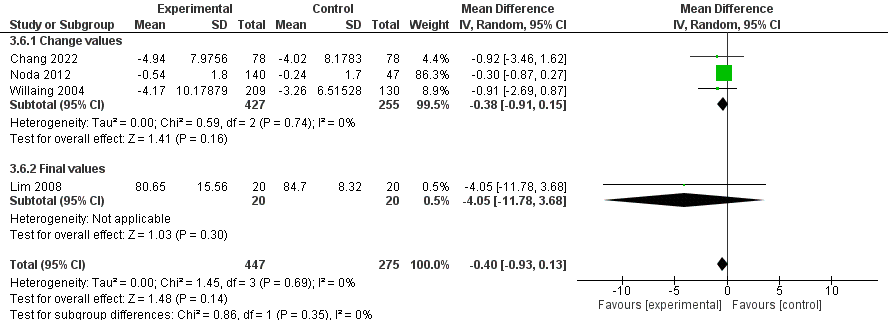

Supplement: nuae149_Supplementary_Data [file nuae149_supplementary_data.docx]
